# Subtle tuning of nanodefects actuates highly efficient electrocatalytic oxidation

**DOI:** 10.1038/s41467-023-37676-6

**Published:** 2023-04-12

**Authors:** Yifan Gao, Shuai Liang, Biming Liu, Chengxu Jiang, Chenyang Xu, Xiaoyuan Zhang, Peng Liang, Menachem Elimelech, Xia Huang

**Affiliations:** 1 State Key Joint Laboratory of Environment Simulation and Pollution Control, School of Environment, Tsinghua University, Beijing, 100084 China; 2 Beijing Key Lab for Source Control Technology of Water Pollution, College of Environmental Science and Engineering, Beijing Forestry University, Beijing, 100083 China; 3 Department of Chemical and Environmental Engineering, Yale University, New Haven, CT 06520-8286 USA

**Keywords:** Electrocatalysis, Electrocatalysis, Synthesis and processing

## Abstract

Achieving controllable fine-tuning of defects in catalysts at the atomic level has become a zealous pursuit in catalysis-related fields. However, the generation of defects is quite random, and their flexible manipulation lacks theoretical basis. Herein, we present a facile and highly controllable thermal tuning strategy that enables fine control of nanodefects via subtle manipulation of atomic/lattice arrangements in electrocatalysts. Such thermal tuning endows common carbon materials with record high efficiency in electrocatalytic degradation of pollutants. Systematic characterization and calculations demonstrate that an optimal thermal tuning can bring about enhanced electrocatalytic efficiency by manipulating the N-centered annulation–volatilization reactions and C-based *sp*^3^/*sp*^2^ configuration alteration. Benefiting from this tuning strategy, the optimized electrocatalytic anodic membrane successfully achieves >99% pollutant (propranolol) degradation during a flow-through (~2.5 s for contact time), high-flux (424.5 L m^−2^ h^−1^), and long-term (>720 min) electrocatalytic filtration test at a very low energy consumption (0.029 ± 0.010 kWh m^−3^ order^−1^). Our findings highlight a controllable preparation approach of catalysts while also elucidating the molecular level mechanisms involved.

## Abstract (web summary)

Achieving controllable fine-tuning of defects is desired for electrocatalysts. Here, the authors present a facile and controllable thermal tuning strategy that enables fine control of nanodefects, thereby endowing common carbon materials with high electrocatalytic oxidation efficiency.

## Introduction

Progress in electrocatalytic science in the past decade has promoted the development of numerous related research areas, including H_2_/O_2_ evolution^1^, CO_2_ reduction^2^, fuel cells^3^, and reactive membrane^4^. A central topic attracting extensive attention in these areas is the development of robust and highly efficient catalysts^5^, which is also the bottleneck that hinders their future advancement and applications. To date, a large number of studies have brought about a variety of electrocatalysts^6–8^, reaching a consensus that the elemental composition and lattice structure of the catalysts have a profound impact on their electrocatalytic performance^9–11^. Correspondingly, achieving controllable fine-tuning of catalysts at the atomic level has become a zealous pursuit^12–14^. However, such fine-tuning or fabrication methods are rarely reported, and the few reported methods are relatively complex and based on scarce and costly materials^15,16^, rendering them undesirable for scaled-up applications.

Recent studies have revealed the fundamental importance of creating structural defects in improving the electrocatalytic performance of catalysts^15,17^, especially for carbon-based catalysts^9,10,18,19^. In general, defects in carbon-based catalysts are caused by the doping of extrinsic substances (e.g., heteroatoms, such as N)^10,18^ or by intrinsic mechanisms which lead to abnormal sites (e.g., vacancy/hole, edge, or topological defect) in conjugated networks^9,20^. The resulting defects can cause significant alteration of electron-density distribution, thereby potentially enhancing electron transfer and electron exchange with exogenous substances and thus forming more reactive catalytic centers^17^. Therefore, an effective approach to fine-tuning catalytic performance can be developed through manipulating the formation of structural defects. While the occurrence of defects seems to be random and uncontrollable^15,16,20^, the lack of theoretical guidance on defect-generation mechanisms hinders the development of fine-tuning methods.

Herein, we propose a thermal tuning strategy that enables fine-tuning of structural defects via subtle manipulation of atomic/lattice arrangements based on a facile and highly controllable carbonization process. Nitrogen-containing polyacrylonitrile (PAN) was employed as a raw material and was electrospun into a porous membrane composed of highly connected nanofibers. Such a nanofiber-based membrane framework has been increasingly recognized as an efficient structure for catalytic processes, owing to its high specific surface area, superior mechanical properties, and versatile application forms^21–23^. After a simple carbonization treatment, the obtained carbon-nanofiber (CN)-based membranes were systematically characterized through X-ray photoelectron spectroscopy (XPS), Raman spectroscopy, and electron spin resonance (ESR), in order to reveal their characteristics in terms of atomic/lattice arrangements. Quantum chemical calculations based on density functional theory (DFT) and multiphysics simulations were performed to further reveal the thermal tuning mechanism. It was demonstrated that fine-tuning of structural defects can be achieved through a simple alteration of the carbonization temperature, thereby conspicuously affecting electrocatalytic performance. Systematic experiments were performed to investigate the detailed electrocatalytic behavior of the CN membranes and the corresponding reaction mechanisms were elucidated. We envision this facile and controllable thermal tuning strategy will lay the foundation for the precise preparation of nitrogen-doped carbon-based catalysts and fine manipulation of their catalytic performance.

## Results

### Controlled thermal treatment enables facile tuning of atomic and lattice arrangements

**Fig. 1:**
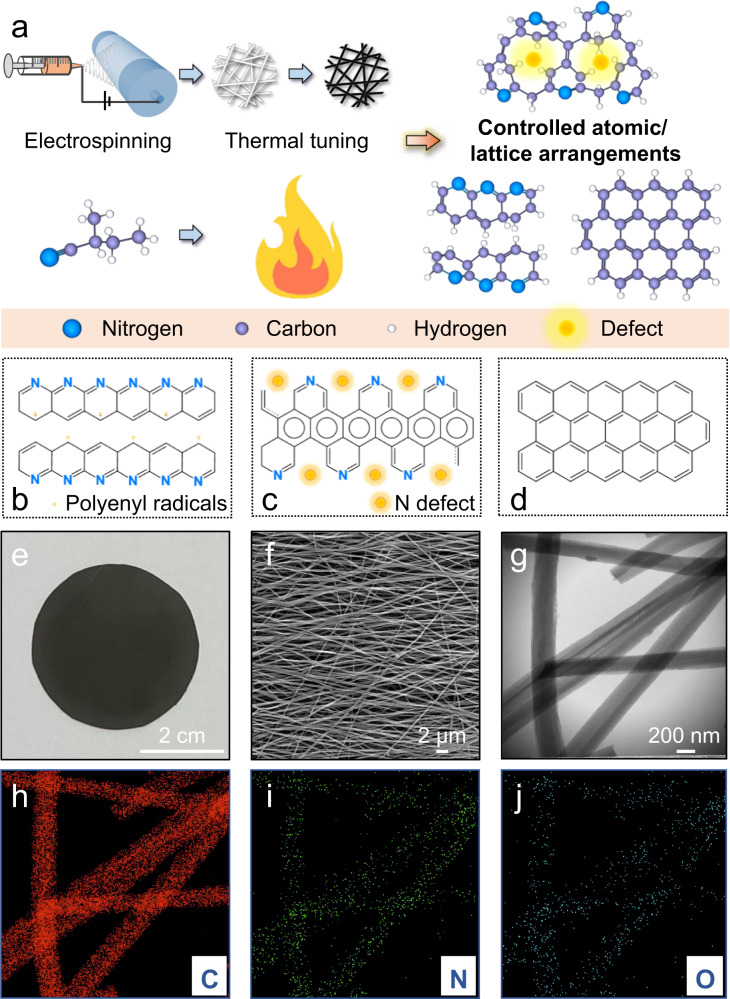
Facile temperature control enables fine-tuning of atomic/lattice arrangements. **a** Schematic protocol of preparation of N-doped carbon nanofiber (CN) membranes and an illustration of the thermal tuning process. Illustrations of the molecular structures of the prepared CNs corresponding to different thermal tuning conditions: **b** insufficient, **c** optimal, and **d** excessive. **e** Photograph, **f** local SEM view, and **g** TEM view of the prepared CN_900 (as a representative) membrane. Elemental mapping images by EDS of the representative CN_900, showing the distributions of **h** (C), **i** (N), and **j** (O), respectively.

An electrospinning–thermal-tuning strategy (Fig. 1a) was employed to fabricate the electrocatalytic CN membranes. The electrospinning process enables the construction of a nanofiber-based framework with a high specific surface area. The thermal tuning process involved a carbonization step to turn the raw nanofiber framework into a conductive carbon-based matrix, while creating reactive sites for reactions. The central idea of the thermal tuning operation is to thermally affect the atomic arrangement of the N atoms contained in PAN and the lattice arrangement of the C matrix for optimized electrocatalytic performance. This thermal tuning operation can be finely controlled through altering the carbonization temperature. A series of CN membranes were fabricated with separate thermal treatments at different temperatures, including 500, 700, 800, 900, 1100, 1300, and 1500°C. The membranes obtained were designated as CN_500, CN_700, CN_800, CN_900, CN_1100, CN_1300, and CN_1500 membranes, respectively.

We have shown that treatment at low-temperature (e.g., 500 °C) can transform the raw PAN into dispersed clusters of locally sintered polypyridine structures^24–27^ (Fig. 1b). A certain number of polyenyl radicals might exist in the resultant materials^24,28^. In contrast, thermal treatment at higher temperatures (e.g., 900 °C) could further promote the linkage of the polypyridine clusters due to enhanced carbonization effect, while creating defects by burning away some N atoms (Fig. 1c). Hence, enhanced electrocatalytic performance can be expected. Further, thermal treatment at excessively high temperature (e.g., 1500 °C) could lead to the re-arrangement of the lattice in the defect areas, resulting in graphene-based structures^29^ (Fig. 1d). Such high-temperature thermal treatment could increase the conductivity, but could also result in reduced electrocatalytic activity. Detailed analyses are discussed in the following sections.

In general, the prepared free-standing CN membranes (see a representative photo for CN_900 in Fig. 1e and more photos in Supplementary Fig. 1) were all composed of highly connected nanofibers (Fig. 1f and Supplementary Fig. 2). Taking CN_900 as a representative, the average diameter of its nanofibers was estimated to be ~237 ± 35 nm based on scanning electron microscope (SEM) and transmission electron microscopy (TEM) images (Fig. 1g). The CN_900 membrane has a porosity of 63.1% and an average pore size of 948.9 nm determined by the mercury intrusion porosimetry method (Supplementary Fig. 3). Therefore, CN_900 membrane belongs to the microfiltration category that can selectively intercept micron-sized particles in water. Besides, the CN_900 membrane optimized by preparation conditions of cylinder rotational speed and external pressure exhibits excellent mechanical properties (Supplementary Fig. 4). The energy-dispersive (EDS) x-ray spectroscopy mapping revealed that C was the most dominant element (Fig. 1h) of the nanofibers, with a trace amount of doped N (Fig. 1i) and O (Fig. 1j).

**Fig. 2:**
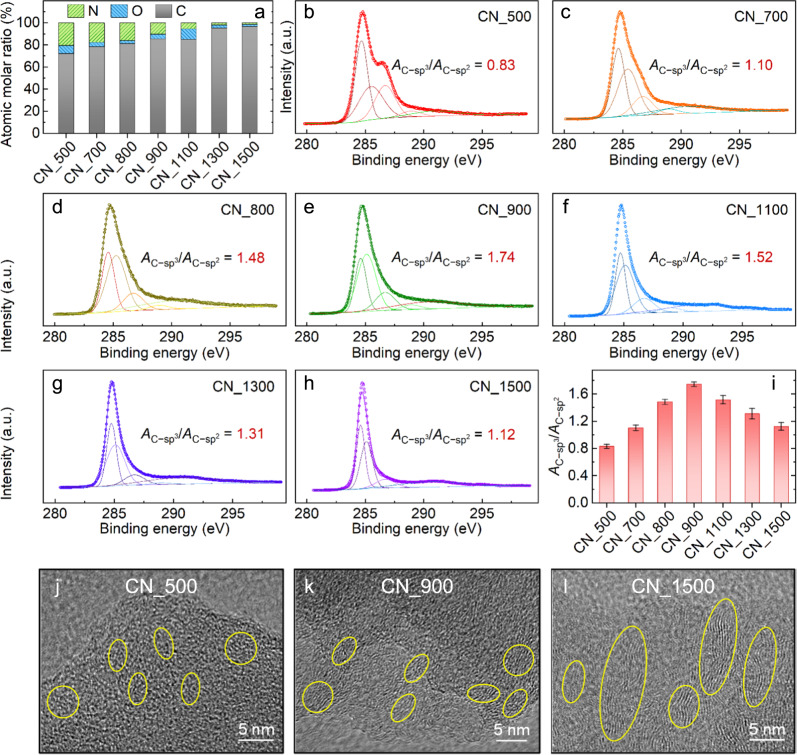
Revealing the variation of elemental constitution and carbon-based lattice. **a** Comparison of the overall surficial elemental composition (by XPS) among the different CNs. High-resolution C 1*s* XPS spectra and fitted peak curves of the **b** CN_500, **c** CN_700, **d** CN_800, **e** CN_900, **f** CN_1100, **g** CN_1300, and **h** CN_1500. **i** Comparison of the calculated *A*_C−sp3_/*A*_C−sp2_ ratios among the different CNs. High-resolution TEM images of the **j** CN_500, **k** CN_900, and **l** CN_1500. The yellow oval areas indicate the disturbed lattice structures. The error bars in panel i represent the standard deviation and were calculated on the basis of at least three experimental data points.

Further characterizations revealed that a simple alteration of the carbonization temperature could effectively lead to changes in the surficial chemical composition and atomic/lattice arrangements of the CNs. The XPS analyses (Fig. 2a) demonstrated that as the temperature increased from 500 to 1500 °C, the proportion of N in the CNs decreased from 20.4% to 1.33%. The same decreasing trend was also observed for O. Fitting analyses of the C 1*s* XPS peaks were further conducted to disclose the change of molecular and lattice structures of the C atoms. As shown in Fig. 2b–h, the dominating peaks at ~284.6 eV and ~285.1 eV are associated with C-*sp*^2^ and C-*sp*^3^, respectively^10,20,30^. The presence of the C-*sp*^3^ configuration normally indicates lattice disorder or defects in carbon materials^10,20,30^. The ratio of the peak intensity of C-*sp*^3^ over C-*sp*^2^ (i.e., *A*_C−sp3_/*A*_C−sp2_) can be used to assess the relative content of the lattice-disordered carbon in the CNs. The calculated *A*_C−sp3_/*A*_C−sp2_ first increased from 0.83 (Fig. 2b, CN_500) to 1.74 (Fig. 2e, CN_900), then gradually dropped to 1.52 (1100 °C), 1.31 (1300 °C) and 1.12 (1500 °C). This change suggested that the disorder degree of C first increased with the rise of temperature, and then decreased when the temperature became excessively high (Fig. 2i). The increase of lattice disorder within the 500–900 °C range was most likely related to the volatilization of N, which results in defects in the carbon matrix. This conclusion was further verified through high-resolution TEM. The observed “river-like” disturbed lattice structures (Fig. 2j–l) and amorphous selected area electron diffraction (SAED) pattern (Supplementary Fig. 5) demonstrated the existence of abundant defects^10^ in the CN_900. The presence of the defective structures was further supported by the observation under high-angle annular dark-field scanning transmission electron microscopy (HAADF-STEM) and annular bright field scanning transmission electron microscopy (ABF-STEM)^10,31,32^ using the focused ion beam-scanning electron microscope (FIB-SEM) technique to prepare the samples (Supplementary Fig. 6). However, the more intensive carbonization at ≥1100 °C resulted in enhanced graphitization with minimal defects remaining (Supplementary Note 1).

The change in the crystal structure of the CNs uncovered by X-ray diffraction (XRD, Supplementary Fig. 7) also agrees with the above finding. The full width at half maximum (FWHM) of the diffraction peak at ~24.9 °C relative to the (002) facet of graphite^20^ usually indicates the degree of crystallization (graphitization). A gradual decrease of the determined FWHM was observed within the 700–1500 °C range. This observation indicates that a higher thermal treatment temperature would lead to a higher degree of crystallization, which is positively correlated with the degree of graphitization.

Further fitting analyses of the XPS N 1*s* peaks revealed more information about the bonding state of the N atoms (Supplementary Fig. 8). As the temperature increased, the areas of both the pyridinic N peak (~398.5 eV) and pyrrolic N peak (~400.0 eV)^33^ decreased. More interestingly, a positive shift was identified for the pyrrolic N peak, indicating its gradual transformation to the graphitic N (~400.9 eV) and thus potentially increased catalytic activity^33^ of CN_900 (Supplementary Fig. 8d). However, the peaks could not be seen in the CN_1300 and CN_1500 spectra, implying that the N atoms were burned off during the treatment.

**Fig. 3:**
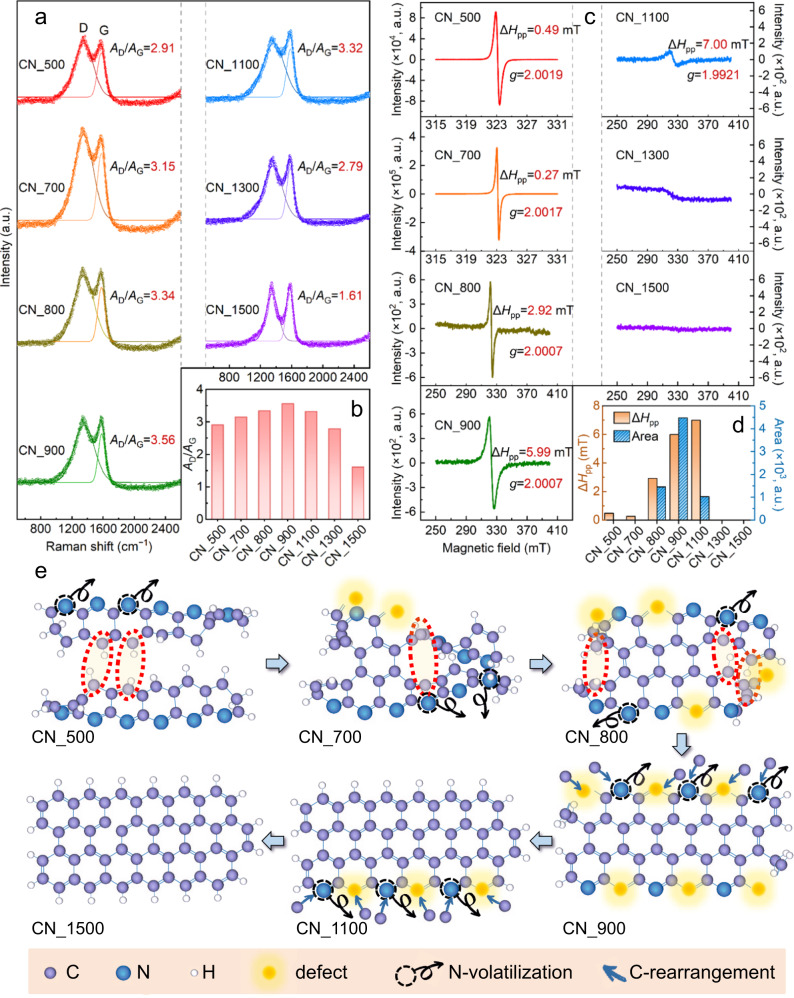
Disclosing the generation, evolution, and annihilation of defects. **a** Raman spectra and fitted peak curves of the CN_500, CN_700, CN_800, CN_900, CN_1100, CN_1300, and CN_1500. **b** Comparison of the *A*_D_/*A*_G_ ratio calculated from Raman spectra. **c** ESR spectra of the different CNs. **d** Comparison of the calculated ESR signal widths (Δ*H*_pp_) and areas among the different CNs. **e** Proposed atomic structure variation during the thermal tuning process, illustrating the gradual annulation (resulting in ring structures), N volatilization (resulting in defects), and graphitization (reducing defects and lattice disorder) processes.

Raman spectroscopy was further employed to reveal the lattice disorder of the CNs (Fig. 3a). Both the D bands (~1360 cm^−1^) associated with the disordered defective C-*sp*^3^ configuration and G bands (~1580 cm^−1^) associated with the ordered graphitic C-*sp*^2^ configuration^10^ were observed for all the CNs. The lattice disorder degree of a carbon material can be evaluated by the ratio of the D band area to the G band area (*A*_D_/*A*_G_)^20^. As presented in Fig. 3b, the calculated *A*_D_/*A*_G_ first increased from 2.91 (CN_500) to 3.56 (CN_900), but then dropped to 1.61 (CN_1500). The variation of the degree of lattice disorder suggested by these results was highly consistent with that revealed by the XPS results (Fig. 2).

The ESR spectra of the CNs (Fig. 3c) revealed the unpaired electron densities, which were associated with the contents of free radicals or lattice defects in the materials. Apparent signal peaks were observed for most CNs, but the signal peaks of CN_500 and CN_700 were totally different from those of CN_800, CN_900, and CN_1100. According to the measured peak-to-peak width (Δ*H*_pp_) data (Fig. 3c, d), the two narrow singlets of CN_500 and CN_700 can be mainly ascribed to the polyenyl radicals (Fig. 1b), which were most likely generated through dehydrogenation during the low-temperature process^24,28^. These polyenyl radicals are commonly recognized as intermediates before the formation of the *sp*^2^ double bonds^24^. However, considering their very limited lifespan in aqueous environments^34^, and their strong tendency to form stable chemical structures via reactions with O_2_, their electrocatalytic activity is negligible^35^. In marked contrast, the broad singlets in the CN_800, CN_900, and CN_1100 spectra are associated with the unpaired electron density of N defects (illustrated in Fig. 1c)^36,37^, which can change the distribution of valence-electron density of the materials^20^ and facilitate electron transfer at the interfacial areas^10^. Notably, it has been widely reported that N-doped defective materials possess high electrocatalytic activity^18,36,38^. Moreover, the integral area of the ESR peak can be used to assess the relative number of defects. As shown in Fig. 3d, the calculated peak area of CN_900 was significantly larger than that of all other CNs, suggesting its potentially superior electrocatalytic performance.

Based on the above discussion, we confirmed that the facile control of the carbonization temperature enabled fine-tuning of the atomic/lattice arrangements during the preparation of the CNs. In general, an optimal thermal treatment (i.e., 900 °C) can generate defects, primarily via the N-centered annulation–volatilization reactions^9,39^. Specifically, the thermal treatment first transforms the precursor PAN (Fig. 1a) into partially polymerized clusters with five-membered and/or six-membered ring structures (e.g., Fig. 1b). Then, volatilization of N takes place, resulting in defects and subsequently increased C-*sp*^3^ configurations (e.g., Fig. 1c). When the temperature is low (e.g., 500 °C), the volatilization process is less significant, resulting in only a few defects. When the temperature is excessively high (e.g., 1500 °C), the raw materials probably go through an annulation–volatilization–graphitization process, also resulting in a few defects (e.g., Fig. 1d). The proposed atomic structure variation during the thermal tuning process is summarized in Fig. 3e.

### Quantum and multiphysics simulations emphasize importance of tuning atomic / lattice arrangements

**Fig. 4:**
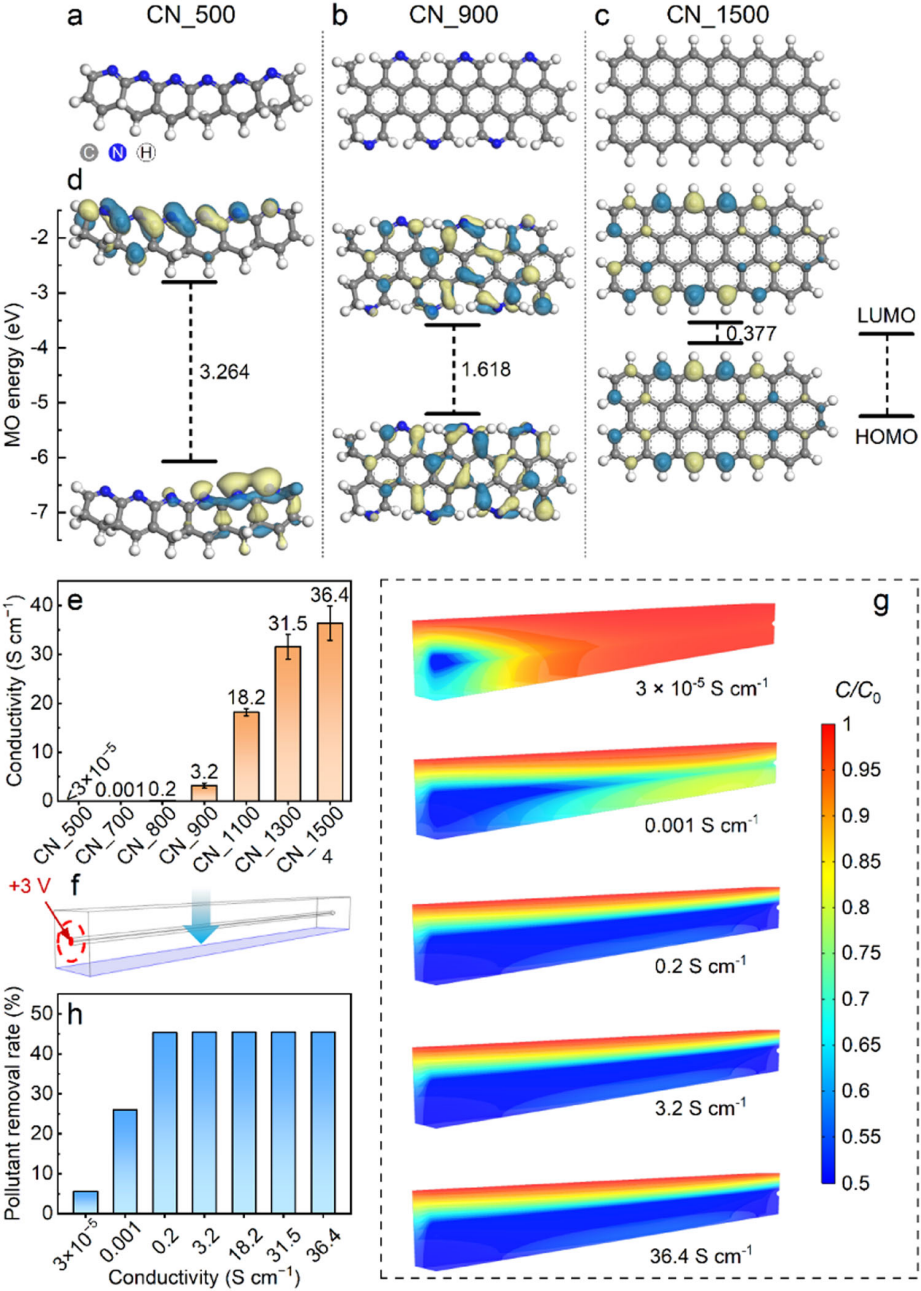
Quantum and multiphysics simulations of electrocatalytic performance. Optimized stable molecular structures, representing the **a** CN_500, **b** CN_900, and **c** CN_1500. **d** Optimized frontier molecular orbitals and LUMO–HOMO energy gaps for the CN_500, CN_900, and CN_1500. **e** Comparison of the measured conductivities among the different CNs. **f** Established model for multiphysics analyses simulating a pollutant solution flowing through a charged CN segment. **g** Computed distributions of the normalized pollutant concentration (*C*/*C*_0_) for different treatment scenarios corresponding to different CN conductivities (i.e., 3 × 10^−5^, 0.001, 0.2, 3.2, and 36.4 S cm^−1^). **h** Comparison of pollutant removal rates (%) at the outlet section calculated on the basis of the simulation results corresponding to different CN conductivities. The error bars in panel e represent the standard deviation and were calculated on the basis of at least three experimental data points.

On the basis of the foregoing results and discussion, three molecular models were established and optimized with DFT, representing CN_500, CN_900, and CN_1500 (Fig. 4a–c). The calculated energies of the highest occupied molecular orbital (HOMO) and lowest unoccupied molecular orbital (LUMO) are demonstrated in Fig. 4d. A smaller HOMO − LUMO gap commonly indicates faster electron transfer^20^. Correspondingly, the calculated HOMO − LUMO gaps followed the order of CN_500 > CN_900 > CN_1500, indicating that electron transfer became faster as the treatment temperature increased. These results corresponded well with the measured conductivities (Fig. 4e). However, as we discuss later, CN conductivity may not be the most important factor affecting its electrocatalytic performance.

The effect of CN conductivity on its electrocatalytic performance was first evaluated through multiphysics simulations. The established multiphysics model (Fig. 4f) simulated a pollutant solution flowing downward through a CN segment in a 3-D simulation domain. According to the computed distributions of normalized pollutant concentration (*C*/*C*_0_, Fig. 4g), the electrocatalytic performance of the CNs behaved differently when the conductivity was lower than 0.2 S cm^−1^. In the CN_500 scenario with the lowest conductivity of 3 × 10^−5^ S cm^−1^, pollutant degradation only occurred at the end section where the bias voltage was applied. The observed weak pollutant degradation in this case is attributable to the hampered electron transfer caused by the low conductivity. When the conductivity increased to 0.001 S cm^−1^, a significant expansion of the space where pollutant degradation occurred was observed, but the degradation still mainly occurred in the vicinity of the end area. The *C*/*C*_0_ decreased along the axial direction, which is linearly related to the decrease of the computed current densities (Supplementary Fig. 9). In marked contrast, a uniform degradation was achieved when the CN conductivity increased to 0.2 S cm^−1^. However, further increase in conductivity (>0.2 S cm^−1^) did not give rise to a higher removal rate (Fig. 4h). These results suggest that CN conductivity could be an important limiting factor for electrocatalytic reactions within the low conductivity range (i.e., <0.2 S cm^−1^), but the effect on electrocatalytic reaction enhancement becomes insignificant in the higher range (i.e., >0.2 S cm^−1^). The results further imply that material conductivity might not be the central pursuit in developing electrocatalysts.

**Fig. 5:**
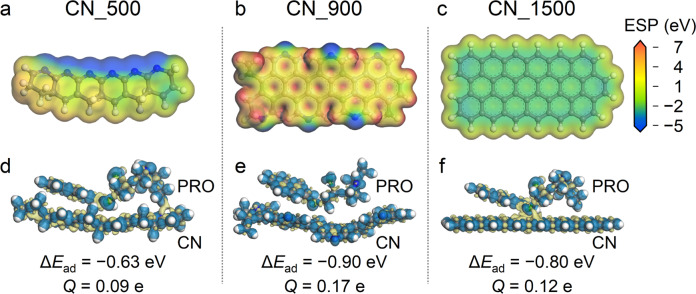
Predicting electrocatalytic activities of different CNs. Distributions of DFT-calculated ESP for the **a** CN_500, **b** CN_900, and **c** CN_1500. Simulations of molecular orbital interactions when a propranolol (PRO) molecule contacts the **d** CN_500, **e** CN_900, and **f** CN_1500, respectively; the calculated adsorption energy (Δ*E*_ad_) and electron transfer number (*Q*) were included.

Additional DFT calculations were performed to support the thermal-tuning mechanism and to predict the impact on electrocatalytic activity. The negative and positive regions in the electrostatic potential (ESP) diagram (Fig. 5a–c) represent the active sites of reduction and oxidation reactions, respectively^40^. In general, the CN_500 and CN_1500 which possessed minor defects were basically negatively charged, except around the carbon matrix and H-edged regions (Fig. 5a, c). In marked contrast, the ESP distribution of CN_900 was much more inhomogeneous (Fig. 5b), suggesting a greater propensity for redox reactions^41^. Additional calculations were also performed based on the Fukui function with Hirshfeld charges, and similar conclusions were reached (see Supplementary Note 2, Supplementary Fig. 10, Supplementary Fig. 11, Supplementary Table 1 and Supplementary Table 2). In addition, according to the Mulliken population analyses of electron density difference (EDD, Fig. 5d–f), the CN_900 exhibited a higher potential for electron transfer than that of the CN_500 and CN_1500. The adsorption energies (*ΔE*_ad_) of a propranolol (PRO) molecule enriched on the CN_500, CN_900, and CN_1500 unit-regions were calculated to be −0.63, −0.896, and −0.80 eV, respectively (Fig. 5d–f). A higher absolute value of *ΔE*_ad_, which is widely recognized as an indication of more lattice defects^42^, indicates a stronger binding affinity and thus higher electrocatalytic efficiency. Additionally, the numbers of electron transfer (*Q*) from the CN_500, CN_900, and CN_1500 to PRO were calculated to be 0.09, 0.17, and 0.12 e, respectively. Overall, CN_900 was expected to achieve the highest electrocatalytic efficiency due to its advantageous defective atomic/lattice arrangements.

### Thermal tuning of atomic/lattice arrangements results in remarkable electrocatalytic performance

The porous structure (porosity of 63.1%) of the electrospun CN membrane allows flow-through configurations, which could facilitate contact with target reactants, increasing reaction opportunities^22^. A great number of studies reported the benefits of fabricating or incorporating electrocatalysts into membrane-like structures, which could be readily used as membrane electrodes in various applications^7,43,44^. Among them, electrocatalytic membrane filtration (EMF) exhibits great potential in tackling the global water crisis, mainly owing to its multifaceted advantages originating from the combination of membrane separation and electrocatalysis. For example, electrocatalytic reactions could be promoted during filtration, owing to abundant active sites due to high porosity, enhanced mass transfer^22^ and confinement effect by membrane nano-channels^45^. In addition, electrocatalysis could also contribute to the mitigation of membrane fouling^21,46^.

**Fig. 6:**
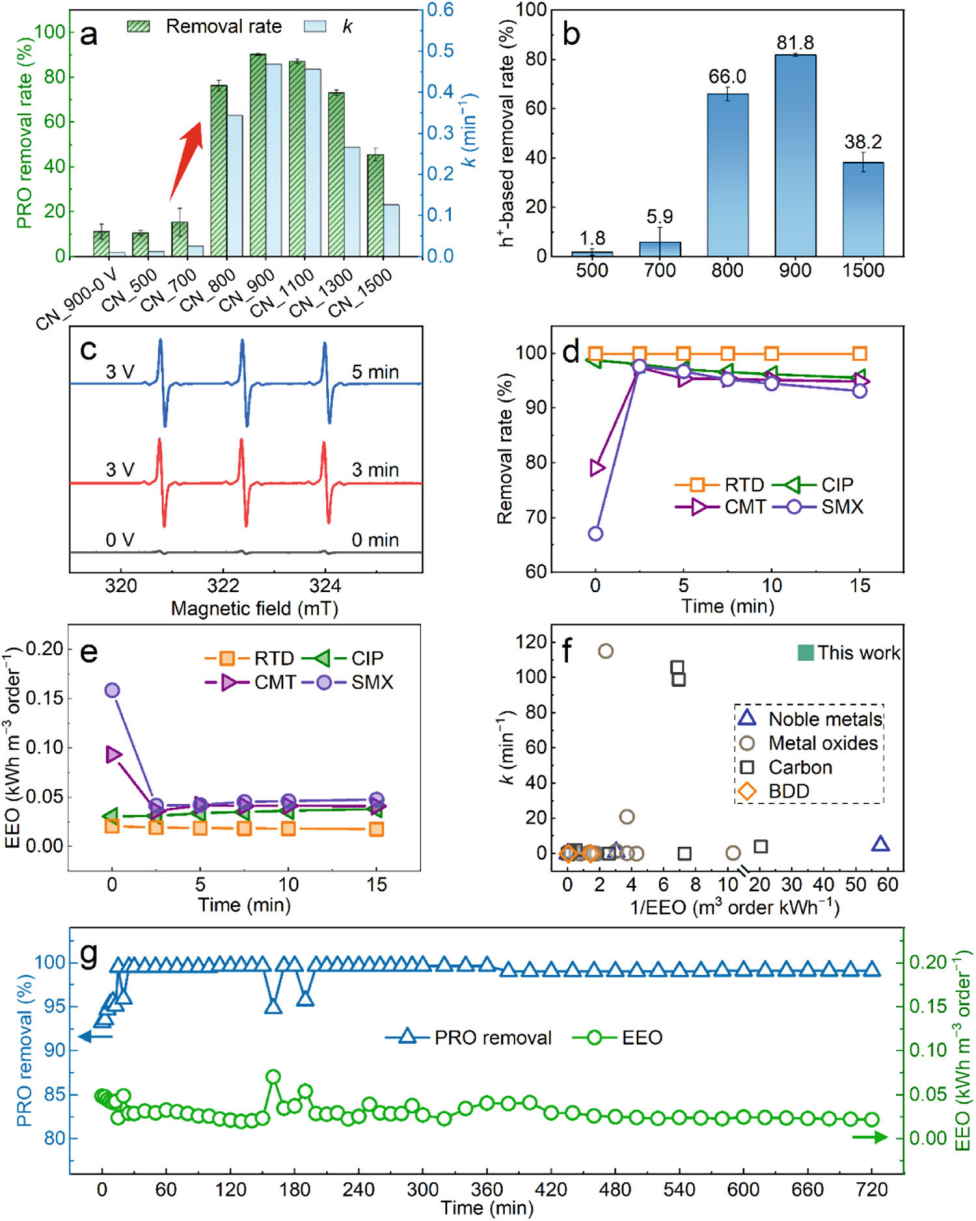
Facile thermal tuning effectively boosts electrocatalytic efficiency. **a** Comparison of the electrocatalytic performance in terms of the PRO removal rate (%) after 5 min treatment in a batch-mode EMF system and kinetic degradation rate constant (*k*, min^−1^, calculated by fitting based on the data in Supplementary Fig. 13b) among the different CN membranes. **b** Comparison of the calculated PRO removal rates owing to h^+^ oxidation among the different CN membranes (more data in Supplementary Fig. 16 and 17). **c** ESR spectra demonstrating the presence of ^1^O_2_ using 100 mM 2,2,6,6‐tetramethyl‐4‐piperidone (TEMP) as the trapping agent based on the CN_900 membrane. Universality evaluation of the performance of CN_900 in terms of (**d**) removal rate and (**e**) electrical energy per order (EEO) in degrading ranitidine (RTD), ciprofloxacin (CIP), cimetidine (CMT), and sulfamethoxazole (SMX) in continuous mode. **f** Comparison of electrocatalytic performance in terms of kinetic rate constant (*k*, min^−1^) and 1/electrical energy per order (1/EEO, m^3^ order kWh^−1^) between this work and other previously published studies (listed in Supplementary Table 3); BDD: boron-doped diamond. **g** Evaluation of the long-term electrocatalytic performance of CN_900 with 500 μg L^−1^ PRO influent at a high water flux of 424.5 L m^−2^ h^−1^ and short retention time of 2.5 s. The error bars represent the standard deviation and were calculated on the basis of at least three experimental data points.

We employed the CN membrane as an anode in a batch-mode EMF system (Supplementary Fig. 12a), and its electrocatalytic performance was systematically evaluated through a series of degradation tests using PRO (20 mg L^−1^) as a model pollutant. As shown in Supplementary Fig. 13a, the CN_500 and CN_700 membranes exhibited a poor degradation performance, only achieving ~15% and ~32% PRO removal after the 15-minute treatment, respectively. In contrast, the CN_800, CN_900, CN_1100, and CN_1300 membranes all achieved >98% removal, although at different degradation rates. These results demonstrated that the high-temperature treatment at >800 °C could successfully boost the electrocatalytic activity, most probably owing to the generated defects (Figs. 2 and 3) and increased CN conductivity (Fig. 4h). Notably, a decrease in removal rate was observed for CN_1500 (85% removal in 15 min), indicating an adverse effect of excessive thermal treatment, likely due to the annihilation of defects at high temperatures. Figure 6a presents a more apparent comparison in terms of the removal rate at 5 min and calculated kinetic degradation rate constant (*k*, min^−1^, based on Supplementary Fig. 13b). The determined electrocatalytic performance followed the order of CN_900 > CN_1100 > CN_800 > CN_1300 > CN_1500 > CN_700 > CN_500, in agreement with the above characterization results. In addition, the excellent electrochemical performance of the CN_900 membrane (Supplementary Fig. 14) is also conducive to its efficient electrocatalytic degradation of pollutants.

It has been widely reported in the literature^47–49^ that N doping of C-based materials is an effective strategy for improving electrocatalytic performance. Here we found that the electrocatalytic performance (in terms of *k*) was not necessarily enhanced with the increase of doped N amount. As shown in Supplementary Fig. 15a, although the CN_500 and CN_700 membranes possessed higher N-doping ratios, their degradation abilities were poor. The chemical state of N is also crucial for electrocatalysis. Previous studies^33,48^ proposed that the improvement of catalytic activity by N doping could be mainly attributed to the formed pyridinic N and graphitic N (Supplementary Fig. 8h). In our study, we proposed an effective thermal tuning approach to the fine manipulation of N states (Supplementary Fig. 8). As the thermal tuning temperature increased from 500 to 900 °C, the pyrrolic N gradually transformed to graphitic N. As the temperature increased above 900 °C, more N atoms were burnt off due to graphitization. Therefore, for the N element in CN, increasing the content of graphitic N and pyridinic N contributes to the enhancement of the electrocatalytic performance. The proposed thermal tuning strategy is effective in controlling both the contents and chemical states of N, thus controlling electrocatalytic performance.

The electrocatalytic degradation tests also verified that CN conductivity was not the only dominating factor. Within the 900–1500 °C range, the higher conductivity (Fig. 4e) did not yield a higher degradation performance but rather resulted in performance deterioration. We note that such deterioration was not observed in the multiphysics simulations (Fig. 4), because the reaction parameters were set to be consistent in all the scenarios. This suggested that the content of lattice defects was an important factor in enhancing the electrocatalytic performance. The correlation analysis between *k* (min^−1^) and *A*_C−sp3_/*A*_C−sp2_ ratio (as an index of the relative lattice defective carbon content) also supported this conclusion (Supplementary Fig. 15b). It is obvious that the electrocatalytic performance and the amount of lattice defective carbon were positively correlated. Only if the content of defective structures was approximately the same, CN membranes with higher conductivity showed the advantage (for example, compare CN_800 and CN_1100).

### Boosted electrocatalytic degradation mainly attributed to h^+^ and ^1^O_2_ oxidation

The previous ESP results (Fig. 5) and characterizations showed that the lattice defects with a greater propensity for redox reactions should be the main active sites for electrocatalysis. Generally, defects can promote the transfer of electrons and the formation of highly oxidative holes (h^+^) in nitrogen-doped carbon materials when excited by external energy (heat, light or bias voltage)^50–52^. Therefore, we surmise that the lattice defects are basically oxidative holes (h^+^)^53^. Accordingly, we performed a series of quenching tests in a batch mode (Supplementary Fig. 12a) with ethylenediaminetetraacetic acid disodium salt (EDTA-2Na, a common quencher for h^+^)^54^ to quantify the contribution of h^+^ to PRO degradation. The obtained results (Fig. 6b, Supplementary Figs. 16 and 17) showed that h^+^ played a dominant role in the electrocatalytic degradation processes. Additionally, furfuryl alcohol (FFA, a quenching agent for singlet oxygen (^1^O_2_)^55^) was used to investigate the possible contribution of ^1^O_2_, which could be generated by the energy conversion or charge transfer during the separation and recombination of electron–hole pairs^52,56,57^. The ESR spectra also clearly showed the typical signals of TEMP-captured ^1^O_2_ (Fig. 6c). Besides, the enhanced degradation of PRO in the deuterium oxide (D_2_O) solvent environment is generally ascribed to the ^1^O_2_-specific kinetic solvent isotope effect^58^ (Supplementary Fig. 18). We observed only a slight increase in *C*/*C*_0_ (Supplementary Fig. 16), indicating a small contribution of ^1^O_2_ to PRO degradation (Supplementary Fig. 19). Probe tests demonstrated that the effect of hydroxyl radical (**·**OH) oxidation was minimal (Supplementary Fig. 20). The applied bias voltage (3 V) may not be sufficient to produce a large amount of effective **·**OH. Furthermore, the measured current densities corresponding to different PRO concentrations followed the order of 20 > 2 > 0.5 mg L^−1^ (Supplementary Fig. 21). Moreover, when the CN_900 was employed as a working electrode in a three-electrode electrochemical system, the addition of PRO resulted in an increase of current density in the linear sweep voltammetry (LSV, Supplementary Fig. 22). Collectively, these results indicated the occurrence of direct electron transfer between the PRO molecules and the membrane, thereby further supporting the major contribution of the direct h^+^ oxidation to the electrocatalytic degradation.

During the EMF process, electrosorption could also lead to a decrease in effluent PRO concentration. To assess this effect, the effluent conductivity during the continuous EMF process was measured (Supplementary Fig. 23). The effluent conductivity remained quite stable, indicating that the effect of electrosorption on PRO removal was negligible.

### Optimized CN membrane exhibits versatile, highly efficient electrocatalytic degradation

The degradation capability of the optimal CN_900 membrane was also verified to be versatile for many other target substances. Ranitidine (RTD), ciprofloxacin (CIP), cimetidine (CMT), and sulfamethoxazole (SMX) were also separately used as representatives of pharmaceuticals and personal care products in the continuous-mode EMF tests. As presented in Fig. 6d, e, high removal rates (>93%) were achieved in all tests, suggesting a great potential for versatile applications. Besides, it was found that the reaction rate of direct anodic oxidation differed for different pollutants (Supplementary Note 3). Further analysis via high-performance liquid chromatography (HPLC)–mass spectrometry (MS)/MS (Supplementary Fig. 24) indicated that most PRO were degraded with few intermediates. Moreover, the acute toxicity analysis revealed that the EMF processes based on the CN_900 membrane could reduce the toxicity of treated water effectively (Supplementary Fig. 25), verifying the environmental friendliness of the CN_900 EMF process.

More importantly, compared with other published studies based on complex materials (noble metals, metal oxides, carbon, and boron-doped diamond (BDD)), the CN membrane showed a remarkably high electrocatalytic efficiency (removal rate > 99% and *k* of 114 min^−1^) with very low energy consumption (0.029 ± 0.010 kWh m^−3^ order^−1^ of EEO) (Fig. 6f and Supplementary Table 3). Moreover, the CN_900 membrane displayed a superior stability in electrocatalytic degradation. Figure 6g presents the PRO removal performance tested for > 720 min at a high flux of 424.5 L m^−2^ h^−1^ (with only 2.4 kPa of applied pressure). Remarkably, no apparent decrease of PRO removal rate was observed. This observation indicated that the membrane was able to continuously degrade >99% of PRO during the flow-through process (~2.5 s contact time). Also, the chemical compositions and morphology of the CN_900 remain stable after the long-term tests (Supplementary Fig. 26), demonstrating a good stability. The CN_900 membrane also has good mechanical durability and remained free-standing after EMF process (Supplementary Fig. 27). The stability of carbon-based electrocatalytic materials is a challenging factor limiting their practical applications. In contrast, the stable operation time of the prepared CN_900 membrane was >4−6 times longer than previously reported^59–61^ carbon-based electrocatalytic membranes (Supplementary Fig. 28 and Supplementary Table 4). Considering that there was still no sign of decline at the end of the long-term test, it appears that CN_900 holds a great potential for long-term practical applications. Moreover, such a degradation process was accomplished at very low energy consumption. Therefore, we demonstrated that high-performance catalysts could be prepared by simple methods based on common materials, without necessarily pursuing rare materials and high-end technologies. Overall, the optimal thermal tuning treatment at 900 °C endowed the CN membrane with a versatile, highly efficient, and long-term stable electrocatalytic degradation capability.

## Discussion

We have shown that the proposed thermal tuning strategy enabled fine-tuning of the atomic/lattice arrangements of both the N element and C matrix. Through simply altering the carbonization temperature, we can control the transformation of pyrrolic N to graphitic N, and at the same time create defects by burning off a portion of N. Simultaneously, alteration of the carbonization temperature would also affect the lattice state of the C matrix. Increasing of temperature first increases the proportion of disordered defective C-*sp*^3^ configurations (beneficial for electrocatalytic reactions), but also changes them back to an ordered graphitic C-*sp*^2^ configuration at high temperatures (e.g., 1500 °C). Consequently, the catalytic performance can be controlled based on the fine-manipulation of atomic/lattice arrangements.

Quantum chemical calculations based on DFT further verified the mechanism of this thermal-tuning strategy. It was demonstrated that an optimal thermal treatment could bring about enhanced redox activity, increased electron transfer capacity, and improved adsorption capability, thus dramatically boosting electrocatalytic efficiency. In combination with multiphysics simulations, the importance of increasing electrocatalytic active sites rather than maximizing conductivity was emphasized.

Benefiting from this tuning strategy, the optimized CN_900 membrane achieved a high electrocatalytic degradation efficiency. It successfully maintained a > 99% degradation rate during a flow-through (~2.5 s contact time), high-flux (424.5 L m^−2^ h^−1^), and long-term (>720 min) test, at a very low energy consumption (0.029 ± 0.010 kWh m^−3^ order^−1^ of EEO), suggesting a great potential for sustainable applications. We envision that this thermal tuning strategy will lay a scientific foundation for future precise and controllable fabrication of various types of catalysts.

## Methods

### Fabrication of CN membranes via electrospinning and thermal tuning

The CN membranes were fabricated with successive electrospinning–thermal-tuning strategy (Fig. 1a). A precursor solution containing 10% (w/v) PAN (*M*_w_ ~150,000, Sigma–Aldrich) in *N*,*N*-dimethylformamide (>99.9%, Shanghai Macklin Biochemical Co., Ltd.) was prepared under vigorous stirring. Then, the solution was introduced to a laboratory electrospinning system (ET-3556H, Ucalery CO., Ltd., China), where the PAN solution was driven by an applied electric field as nanofibers to a rotating (2500 rpm) cylinder collector. The tip voltage, collector voltage, inner needle diameter, tip-to-collector distance, and injection speed during electrospinning were set to 15 kV, −7.5 kV, 0.5 mm, 15 cm, and 1.5 mL h^−1^_,_ respectively. After 10 h of electrospinning at 40% ± 10% humidity and 25 ± 5 °C, the obtained PAN nanofiber mat was detached for the subsequent thermal tuning treatment. For thermal tuning, the nanofiber mat was first pre-oxidized in air at 280 °C for 2 h with a heating rate of 2 °C min^−1^, and then subjected to a higher temperature (i.e., 500, 700, 800, 900, 1100, 1300, or 1500 °C) heating treatment in Ar for 1 h with a heating rate of 5 °C min^−1^. This thermal tuning treatment was intended to create defects via carbonization and maximizing the defect activity via shaping atomic and lattice arrangements. After cooling, the resulting membranes were used for characterization and designated as CN_500, CN_700, CN_800, CN_900, CN_1100, CN_1300, and CN_1500 membranes, respectively. Furthermore, when the carbonization operation for CN_900 was performed in an air atmosphere with O_2_, the obtained material would not have a defective structure (Supplementary Fig. 29a), and would not be free-standing due to its poor mechanical strength (Supplementary Fig. 29b). On the one hand, the inert Ar atmosphere promotes the volatilization of N and C-based *sp*^3^/*sp*^2^ configuration alteration; on the other hand, the Ar atmosphere prevents the carbon structure from being destroyed by the oxidation of oxygen in the air.

### Characterizations of CN membranes

Membrane morphology was observed through SEM (Zeiss Merlin Compact, Germany) and TEM (JEM-2100F, Japan). Elemental distribution and chemical composition of the membrane were determined through TEM mapping and XPS (ESCALAB 250Xi, Thermo Scientific, England), respectively. Membrane crystal structure was investigated with XRD (D8/Aduance, Bruker, Germany). Membrane molecular structure was evaluated with a Raman spectrometer (LabRAM HR Evolution, Horiba Co., Ltd., France) at 532 nm. An X-band ESR spectrometer (JES-FA200, JEOL, Japan) was employed to reveal the defective structures. The peak-to-peak width of the singlet in the obtained ESR spectra was recorded as Δ*H*_pp_ (mT). Membrane conductivity was assessed with a four-probe tester (280SJ, Four Dimensions, USA).

### Quantum chemical calculations based on DFT

The Gaussian 16 C. 01 software was employed for all the model constructions and calculations. The ideal molecular structures of the representative materials (i.e., CN_500, CN_900, CN_1500, and PRO) were first constructed with their edges terminated by H atoms to maintain electroneutrality. Optimizations of the molecular structures were performed using the IEFPCM base model with the B3LYP functional and 6-311 G (d, p) basis set. The calculation accuracy can be improved by correcting the weak interaction with the DFT-D3 method. For all the geometric relaxations, the selected convergence indicators were 1.0 × 10^−4^ Ha/Å for the total energy, <0.02 Ha/Å for the force on each atom, 0.05 Å for the tolerance deviation, and 5.0 × 10^−3^ Ha/atom for the self-consistent accuracy. Then the ESP diagram was drawn based on the calculation results to analyze the relative electron density in the molecules. All calculations were performed under ground-state geometric conditions. The results of EDD were achieved by Miliken Population Analysis. The electron transfer number (*Q*) was obtained from the EDD results. The adsorption energy (Δ*E*_ad_) was calculated from

1 $$\triangle {E}_{{{\rm{ad}}}}={E}_{{{\rm{tot}}}}-\left({E}_{{{\rm{cm}}}}+{E}_{{{\rm{ads}}}}\right).$$

where the *E*_tot_, *E*_cm_, and *E*_ads_ indicated the total energy of the adsorption matrix, CN membrane, and adsorbates, respectively.

### Modelling and simulation of pollutant degradation behavior

The microscopic model of a CN (Fig. 4f) was established using the COMSOL Multiphysics 5.6 software to investigate the effect of conductivity change due to thermal tuning on its electrocatalytic behavior. The established model imitates a pollutant-containing solution flowing downward through a CN with a diameter of 240 nm and a length of 20 µm in a 3-D simulation domain. The reference electrode was placed on the lower surface. A bias voltage of 3 V was applied to the left port of the CN. Different simulations were separately performed corresponding to different CN conductivities, including 3 × 10^−5^, 0.001, 0.2, 3.2, 18.2, 31.5, and 36.4 S cm^−1^, which were determined according to the actual conductivity measurements of the CN_500, CN_700, CN_800, CN_900, CN_1100, and CN_1500 membranes, respectively. In the simulations, the distributions of normalized pollutant concentration (*C*/*C*_0_) within the 3-D domain were calculated. The distributions of electric current density and other electric parameters were solved by the Poisson equation^62^. The advection and diffusion of pollutant were described by combining the Navier-Stokes and Nernst-Planck equations^63^. The boundary condition and related coefficients^64^ were listed in Supplementary Table 5.

### Evaluation of electrocatalytic performance

The electrocatalytic performance of the CN membranes was assessed through a set of filtration degradation experiments using an EMF system comprising an EMF reactor, a peristaltic pump, a reservoir beaker, and an external power (SS-L303SPD, ABF, China). The membrane to be tested and a titanium mesh (Zhuosheng Wire Mesh Co., Ltd., China) were installed in the EMF reactor as the anode and cathode, respectively. The effective filtration area was 7 cm^2^. A synthetic solution containing PRO (hydrochloride, Shanghai Macklin Biochemical Co., Ltd., China; representing micropollutants, 20 mg L^−1^ unless specified) and 10 mM Na_2_SO_4_ (as electrolyte) was used as feed. During the test, a 3 V voltage was applied to the EMF reactor to initiate electrocatalytic reactions. For different research purposes, the EMF system can be operated in either a batch mode (Supplementary Fig. 12a) or a continuous filtration mode (Supplementary Fig. 12b). The batch mode was adopted for the investigation of the effect of thermal treatment on defects and degradation mechanism. The continuous mode was adopted for the evaluation of effluent pH (Inlab 731-ISM, Mettler Toledo, USA) / conductivity (Inlab Expert Pro-ISM, Mettler Toledo, USA) variations and long-term degradation stability.

PRO concentrations (denoted as *C*) were determined with a high-performance liquid chromatograph (HPLC) instrument (Agilent 1200) equipped with a C-18 column (Agilent Eclipse XDB-C18). A mixture containing 7:3 (v/v) 0.1% phosphoric acid solution/acetonitrile was used as the eluent. The injection volume was set to 5 μL when the feed PRO concentration was 20 mg L^−1^, and set to 50 μL when that was 0.5 mg L^−1^. The flow rate, column temperature, and detection wavelength were set to 1.0 mL min^−1^, 30 °C, and 218 nm, respectively. The chemical composition of the effluent was further analyzed with an HPLC‒MS/MS (Agilent, US) instrument^22^. The luminescent bacteria *Vibrio fischeri* was used for the acute toxicity analysis (ISO 11348-3 as reference)^65^. The acute toxicity test relies on the inhibition of cell activity to decrease the respiratory rate, which further leads to the corresponding decrease of bacterial luminescence.

The rate constant *k* of PRO degradation was calculated by fitting the data to pseudo-first-order kinetics:

2 $${{{{{\rm{ln}}}}}}\frac{C}{{C}_{0}}-{kt}.$$

where *C*_0_ (mg L^−1^) and *C* (mg L^−1^) are the initial and timely measured (corresponding to *t*, min, indicating the time duration after the voltage was applied) PRO concentrations, respectively. The EEO (kWh m^−3^·order^−1^), as an energy consumption index, was calculated as:

3 $${EEO}=\frac{{UI}\frac{t}{60}\times {10}^{-3}}{V{\log }_{10}\left(\frac{{C}_{0}}{C}\right)}.$$

where the *U* (V) is the applied voltage, *I* (A) is the measured current, *t* (min) is the reaction time, and *V* (m^3^) is the total influent volume involved in the reactions.

### Revealing mechanisms of electrocatalytic reactions

A series of quenching, ESR and probe tests were conducted to identify the presence of multiple reactive species (e.g., h^+^, ^1^O_2_) and quantify their contributions to the PRO degradation during the EMF tests. For the quenching tests, a 4 mM portion of EDTA-2Na (Sinopharm Chemical Reagent Co., Ltd., China) and a 16 mM portion of FFA (Shanghai Macklin Biochemical Co., Ltd., China) were separately added to the feed solution (20 mg L^−1^ PRO, 10 mM Na_2_SO_4_) to quench the possibly generated h^+^ and ^1^O_2_, respectively. For the ESR tests, a 100 mM portion of TEMP (Meryer Co., LTD., China)^66^ was added as the trapping agent of ^1^O_2_ to a 10 mM Na_2_SO_4_ solution, which was then used as the feed for the EMF experiment. The effluent was analyzed using an X-band ESR spectrometer (JES-FA200, JEOL, Japan). The adopted recording parameters were 1 mW for microwave power, 15 mT for sweep width, 0.1 mT for modulation amplitude, and 120 s for sweep time. Probe tests were conducted to identify the presence of **·**OH^7^. A trace amount of TA (100 *μ*M) was added to a 10-mM Na_2_SO_4_ solution to capture **·**OH, which was then changed to hTA.

## Supplementary information

Supplementary Information

## Data Availability

The data generated in this study are provided in the main text and/or Supplementary information, where the source data are listed in the Source Data file. Extra data are available from the corresponding authors upon reasonable request. Source data are provided with this paper.

## Source data

Source Data
