# Supplementary material for: Subtle tuning of nanodefects actuates highly efficient electrocatalytic oxidation
Source: Nat Commun. 2023 Apr 12;14:2059. doi: 10.1038/s41467-023-37676-6 (PMC10097648; doi:10.1038/s41467-023-37676-6)
Supplement: Supplementary file 1 — Supplementary Information [file 41467_2023_37676_MOESM1_ESM.pdf]

Supplemental Information

Subtle tuning of nanodefects actuates highly efficient electrocatalytic oxidation

Yifan Gao[a, 1], Shuai Liang[b, 1*], Biming Liu[a], Chengxu Jiang[a], Chenyang Xu[a], Xiaoyuan Zhang[a], Peng Liang[a], Menachem Elimelech[c*], Xia Huang[a*]

[a]State Key Joint Laboratory of Environment Simulation and Pollution Control, School of Environment, Tsinghua University, Beijing 100084, China

[b]Beijing Key Lab for Source Control Technology of Water Pollution, College of Environmental Science and Engineering, Beijing Forestry University, Beijing 100083, China

[c]Department of Chemical and Environmental Engineering, Yale University, New Haven, CT 06520-8286, USA

*Corresponding author: E-mail: xhuang@tsinghua.edu.cn;

*Corresponding author: E-mail: shuai_liang@bjfu.edu.cn

*Corresponding author: E-mail: meachem.elimelech@yale.edu

[1]Y.G. and S.L. contributed equally to this work

This PDF file includes:

Supplementary Note 1 to 3
Supplementary Figures 1 to 29
Supplementary Tables 1 to 5

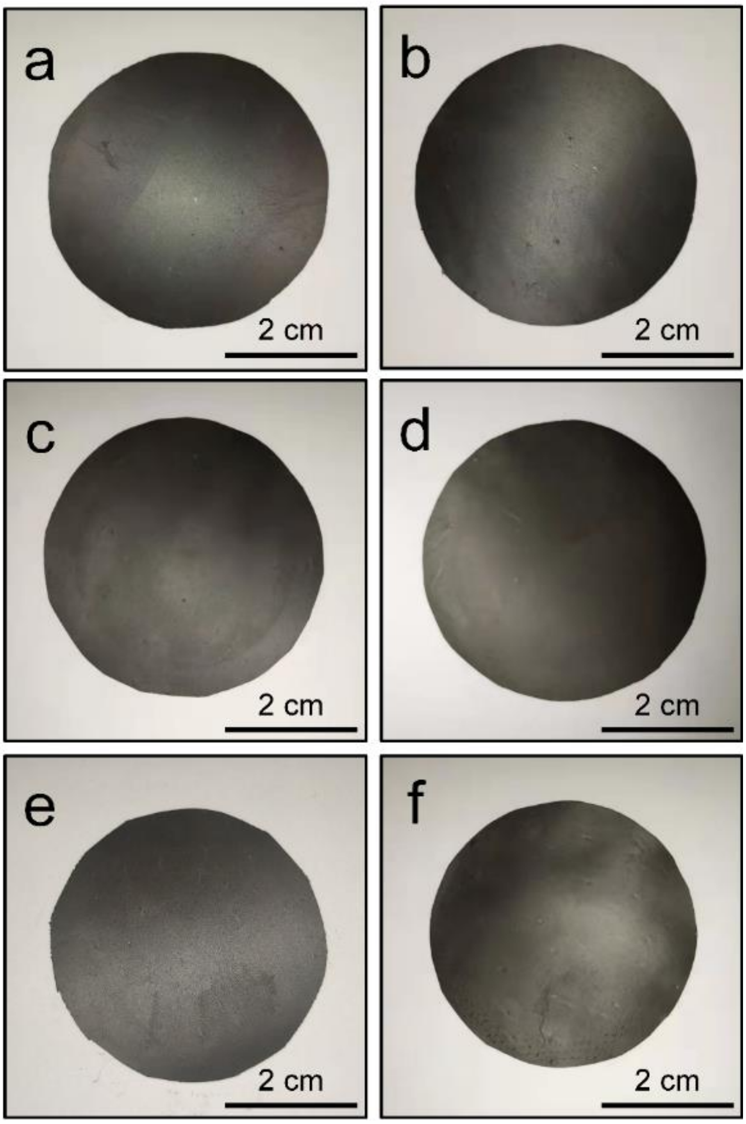

Supplementary Figure 1. Photographs of the different CN membranes (i.e., (**a**) CN_500, (**b**) CN_700, (**c**) CN_800, (**d**) CN_1100, (**e**) CN_1300, and (**f**) CN_1500).

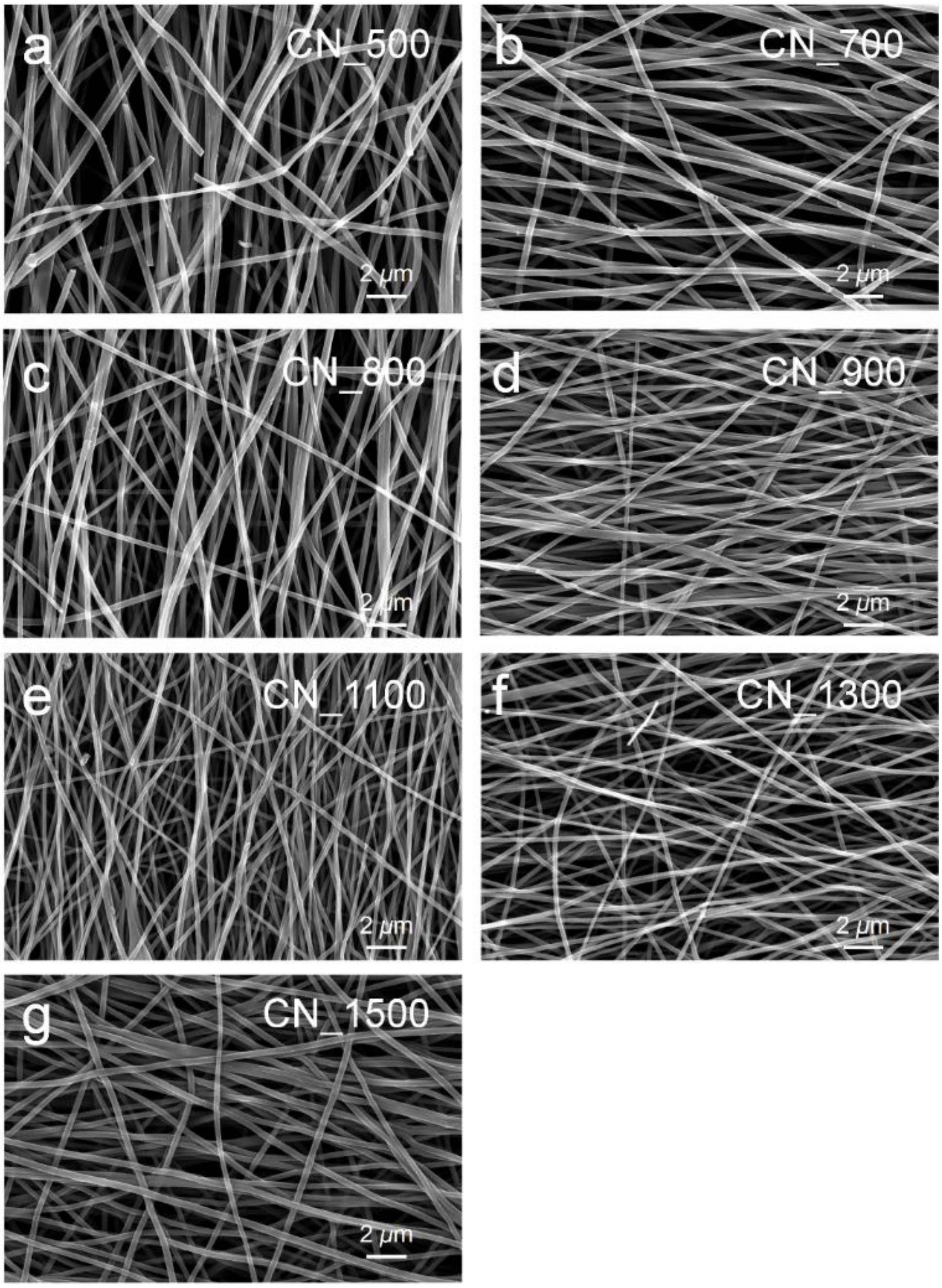

Supplementary Figure 2. SEM images of the (**a**) CN_500, (**b**) CN_700, (**c**) CN_800, (**d**) CN_900, (**e**) CN_1100, (**f**) CN_1300, and (**g**) CN_1500 membranes.

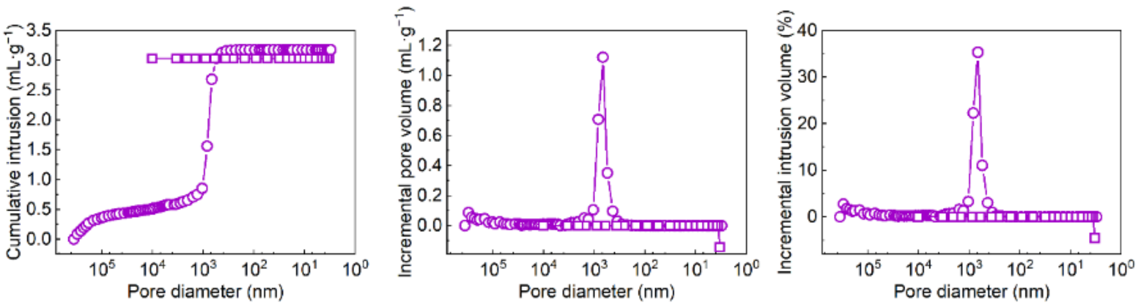

Supplementary Figure 3. Results of the mercury intrusion porosimetry method for the prepared CN_900 membrane.

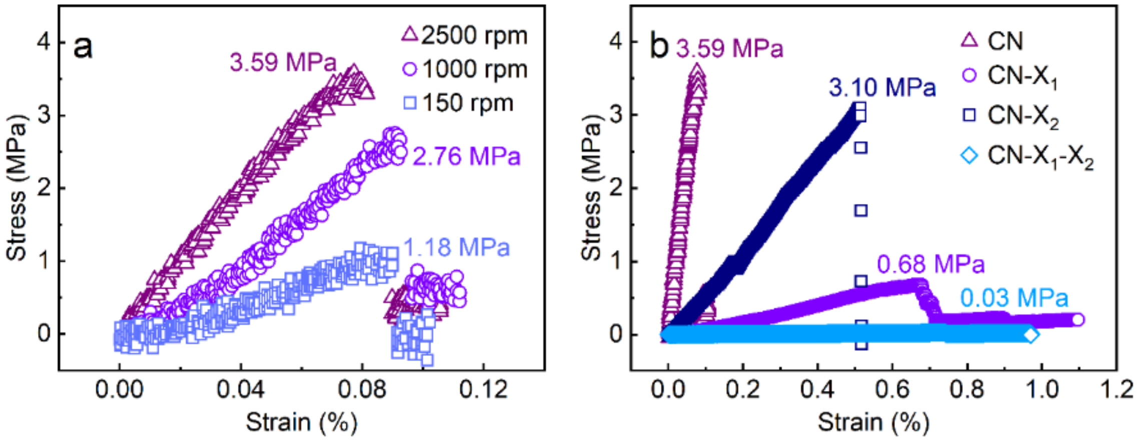

Supplementary Figure 4. Measured tensile stress–strain curves of different CN membranes prepared with (**a**) different cylinder rotation speed and (**b**) different external pressure conditions; the suffixes X_1 and X_2 indicate the absence of the pre-oxidation pressure and the absence of the carbonization pressure, respectively. Note: all the CN membranes in this figure was carbonized at 900 °C.

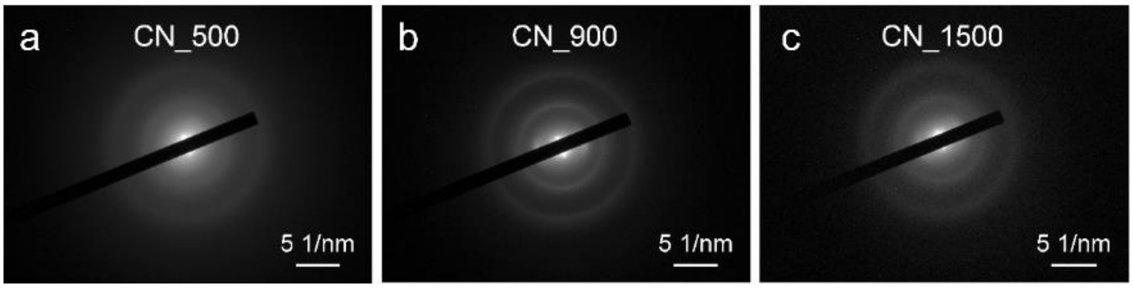

Supplementary Figure 5. Selected area electron diffraction (SAED) patterns of the (**a**) CN_500, (**b**) CN_900, and (**c**) CN_1500.

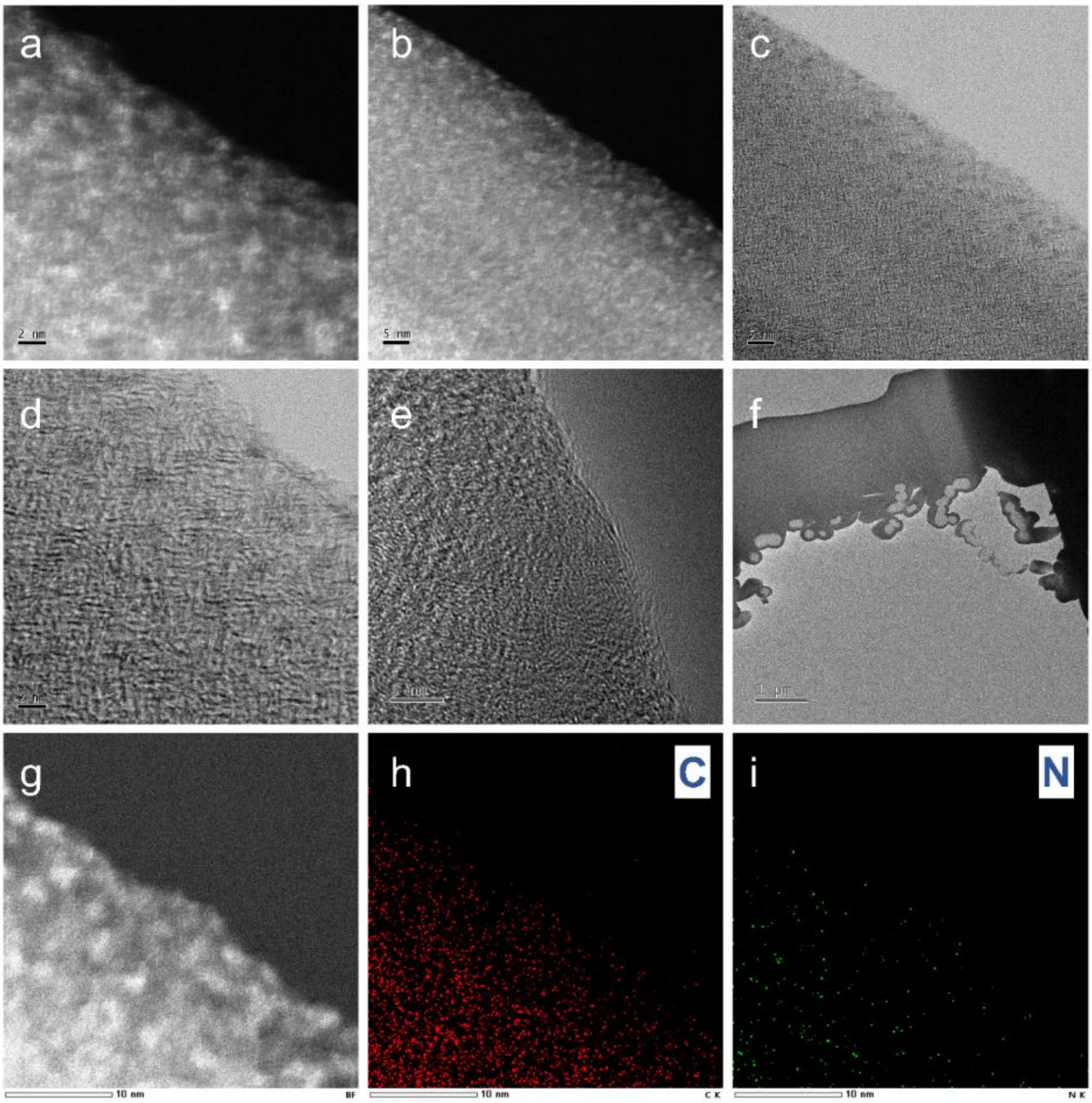

Supplementary Figure 6. (**a** and **b**) High-angle annular dark-field scanning transmission electron microscopy (HAADF-STEM) images of CN_900. (**c** and **d**) Annular bright field scanning transmission electron microscopy (ABF-STEM) images of CN_900. (**e**) HRTEM image of CN_900. (**f**) TEM image showing the CN_900 prepared by focused ion beam-scanning electron microscope (FIB-SEM) technique. (**g–i**) Elemental mapping images by EDS of CN_900.

Supplementary Note 1.

The “river like” disturbed lattice structures could hardly be observed in CN_500 (**Figure 2j**), indicating that the graphitization of CN_500 was negligible. As for CN_1500, there were more obvious and longer lattice stripes (**Figure 2l**) with rare amorphous structures. This suggested that the graphitization degree of CN_1500 was the highest, leaving few defective structures.

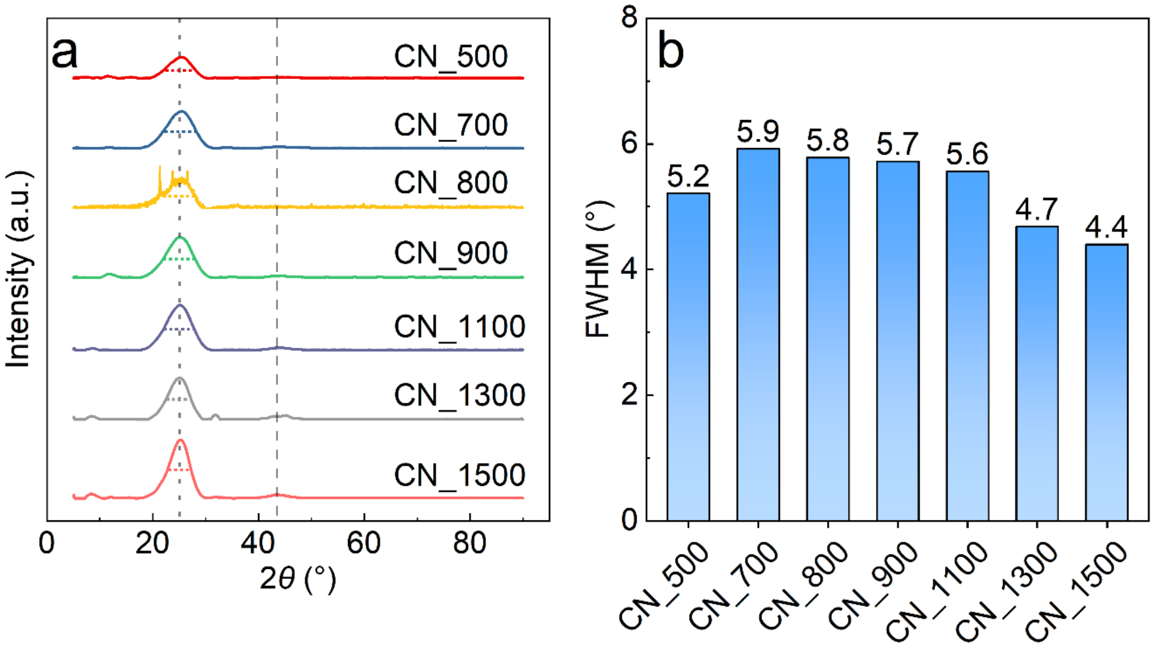

Supplementary Figure 7. (**a**) XRD patterns and (**b**) determined full width at half maximum (FWHM) data of the CN_500, CN_700, CN_800, CN_900, CN_1100, CN_1300, and CN_1500.

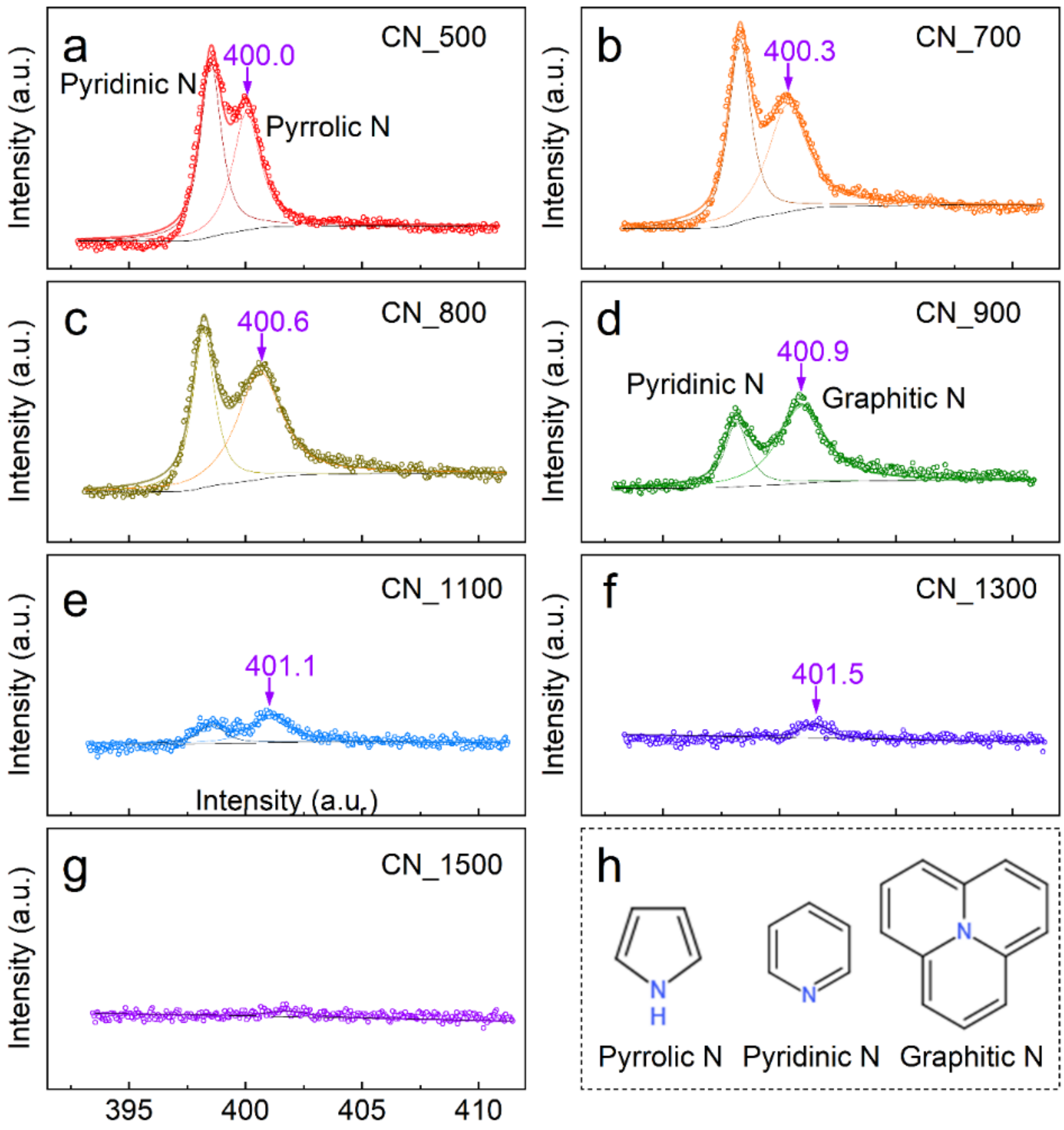

Supplementary Figure 8. High-resolution *N 1s* XPS spectra and fitted curves of the (**a**) CN_500, (**b**) CN_700, (**c**) CN_800, (**d**) CN_900, (**e**) CN_1100, (**f**) CN_1300, and (**g**) CN_1500; and (**h**) chemical structures of pyrrolic N, pyridinic N, and graphitic N.

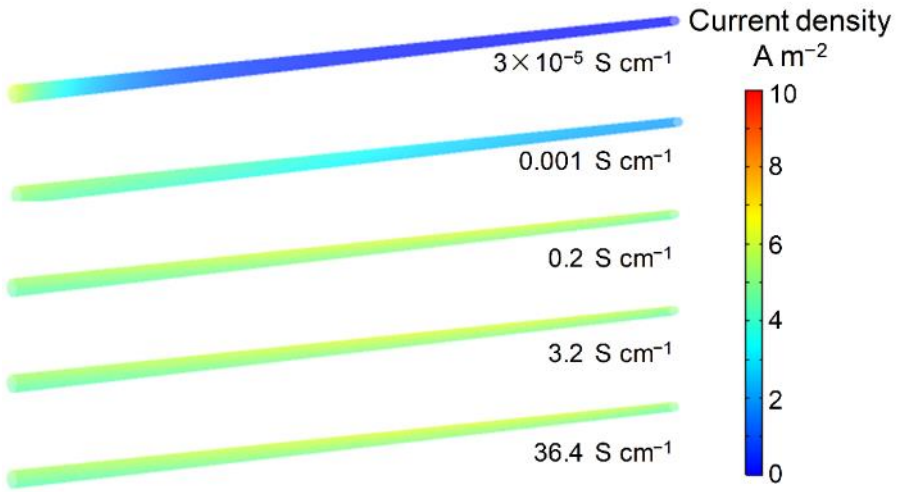

Supplementary Figure 9. Computed distributions of current density (A m^{-2}) on the CN models in different simulation scenarios (**Figure 5**, i.e., at different CN conductivities: 3×10^{-5}, 0.001, 0.2, 3.2, and 36.4 S cm^{-1}).

Supplementary Note 2

The analysis based on Fukui function was used to verify the region of active sites[1] of CNs as well as to predict electrophilic, nucleophilic, and free radical attacks in pollutant regions[2]. The program Multiwfn[3,4] was used to calculate the Fukui function with Hirshfeld charges. As shown in **Supplementary Fig. 10**, the results of the Fukui function analysis of different CNs are similar to the previous DFT calculations results. The f^- distribution of CN_900 was much more inhomogeneous. The regions around the defects have larger values of f^- (**Supplementary Table 1**), which means that these regions are more reactive. The electrophilicity indices of CN_500, CN_900, and CN_1500 unit-regions were calculated to be 1.72, 2.84, and 2.49 eV, respectively. Overall, CN_900 was expected to achieve the highest electrocatalytic reactivity based on the Fukui function analysis. In addition, the Fukui function analysis (**Supplementary Fig. 11** and **Supplementary Table 2**) shows that the amino group (N4) in the PRO structure are more susceptible to electrophilic attack (higher f^-) and the benzene ring (C10–13, C15–18) is highly susceptible to free radical attack (higher f^0).

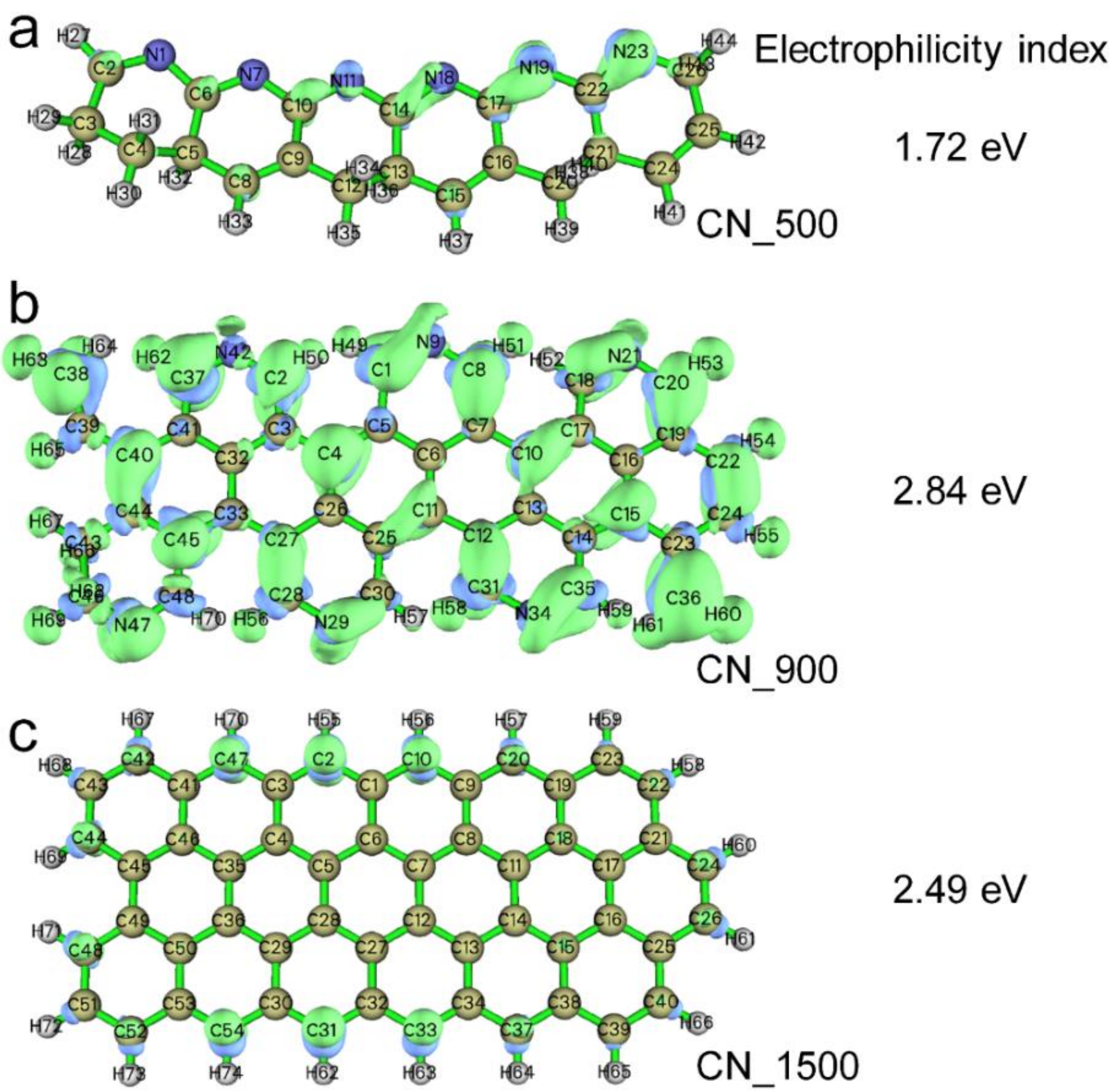

Supplementary Figure 10. Representation of the Fukui function f⁻ of the (a) CN_500, (b) CN_900, and (c) CN_1500. The calculated electrophilicity indices were included.

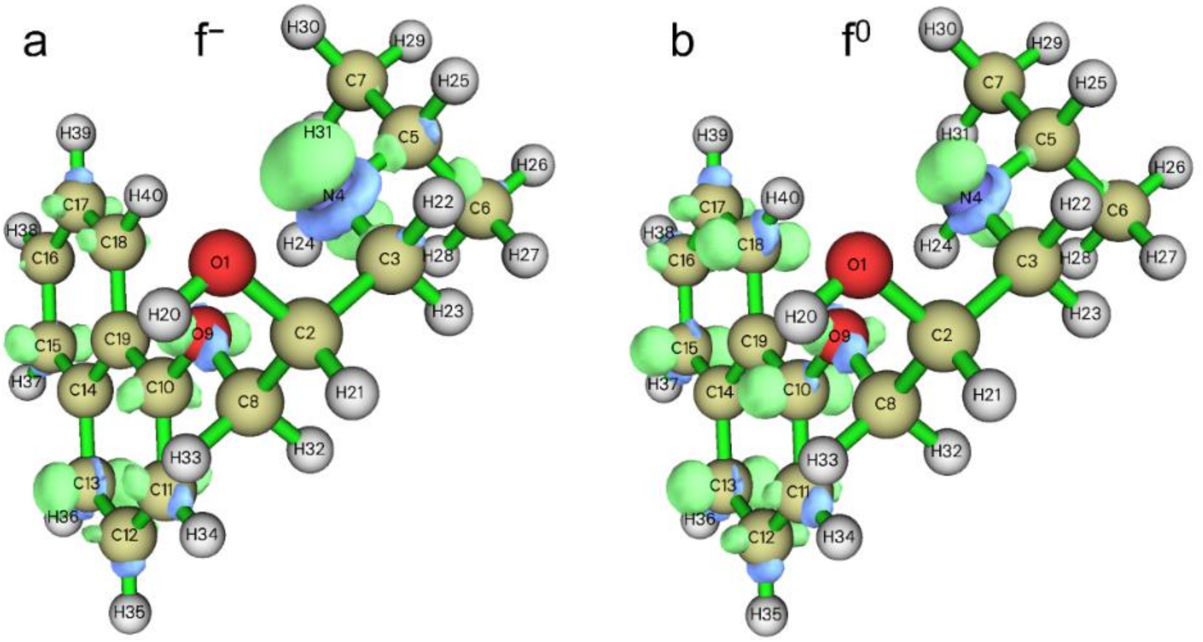

Supplementary Figure 11. Representation of the (a) Fukui function f⁻ and (b) Fukui function f⁰ of the PRO.

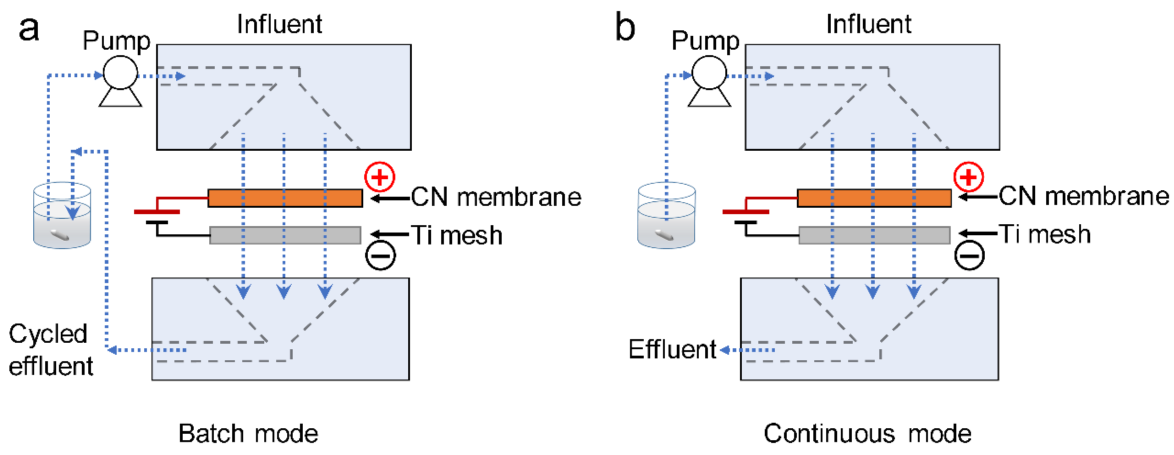

Supplementary Figure 12. Configurations of the electrocatalytic membrane filtration (EMF) system operated at the (**a**) batch mode (i.e., effluent cycles back to feed reservoir, from where the samples are taken for analyses) and (**b**) continuous mode (i.e., feed solution flows one time through the reactor without circulation and the effluent is collected for analyses).

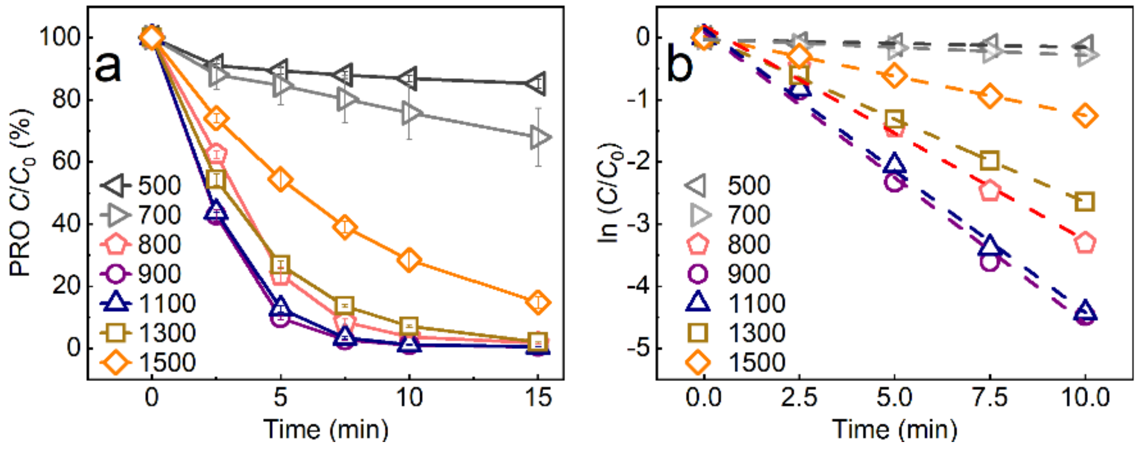

Supplementary Figure 13. Comparison of the electrocatalytic performance in a batch-mode EMF system among the different CN membranes (i.e., CN_500, CN_700, CN_800, CN_900, CN_1100, CN_1300, and CN_1500) in terms of the (**a**) C/C_0 and (**b**) $\ln(C/C_0)$ decline trends. The error bars represent the standard deviation and were calculated on the basis of at least three experimental data points.

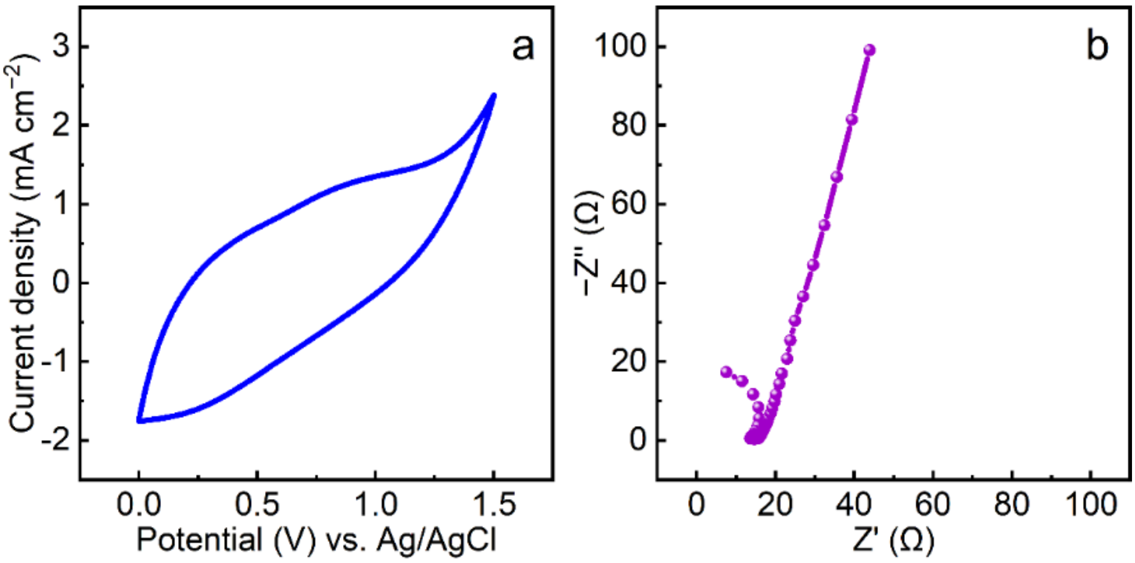

Supplementary Figure 14. (**a**) Cyclic voltammogram and (**b**) electrochemical impedance spectroscopy of the CN_900 membrane.

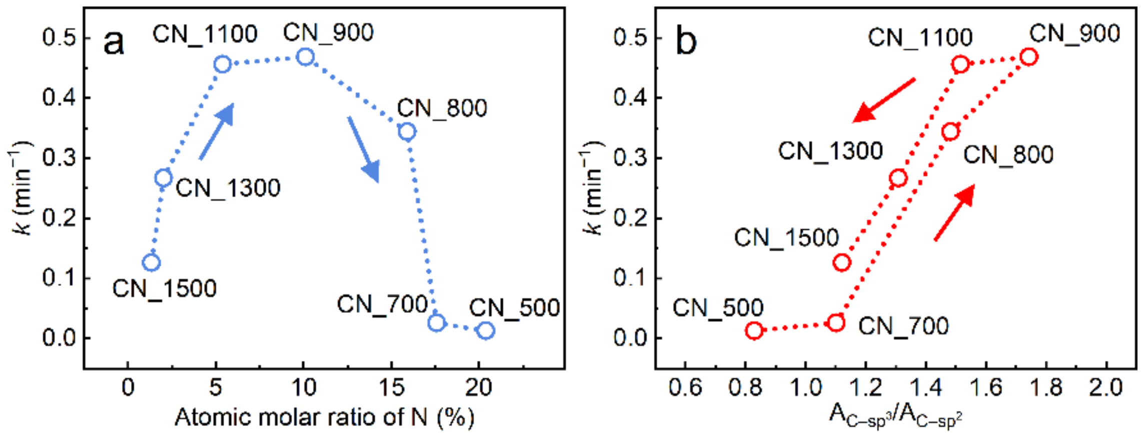

Supplementary Figure 15. (**a**) Correlation analysis between the calculated kinetic constants (*k*, min^{-1}) of PRO degradation in a batch mode and atomic molar ratios of N for the different CN membranes (i.e., CN_500, CN_700, CN_800, CN_900, CN_1100, CN_1300, and CN_1500). (**b**) Correlation analysis between *k* (min^{-1}) and A_{C-sp3}/A_{C-sp2} ratio for the different CN membranes.

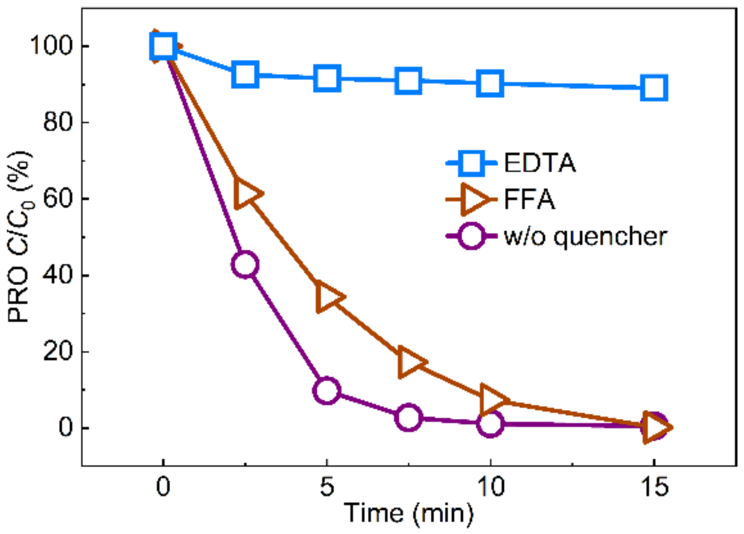

Supplementary Figure 16. Quenching tests revealing the contribution of direct oxidative hole (h^+, quenched by 4 mM EDTA-2Na) and singlet oxygen ($^1O_2$, quenched by 16 mM FFA) to the degradation of PRO (20 mg L^{-1}) by the CN_900 membrane.

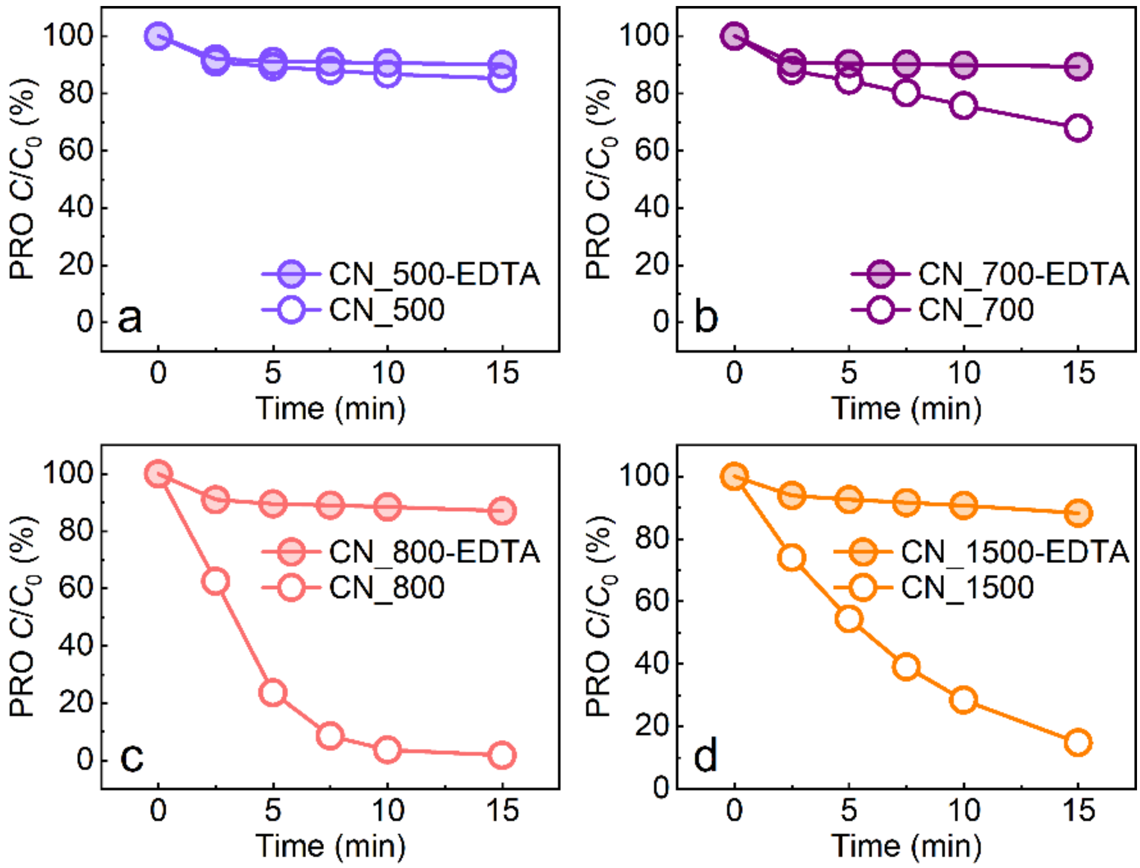

Supplementary Figure 17. Quenching tests revealing the contribution of direct oxidative hole (h^+, quenched by 4 mM EDTA-2Na) to the degradation of PRO (20 mg L^{-1}) for different CN membranes: (**a**) CN_500, (**b**) CN_700, (**c**) CN_800, and (**d**) CN_1500.

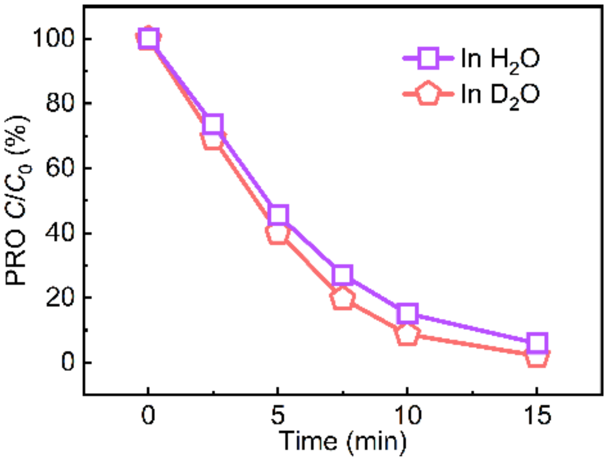

Supplementary Figure 18. Comparison of PRO (50 mg L^{-1}) degradation by CN_900 electrocatalytic membrane in H_2O and D_2O solvents.

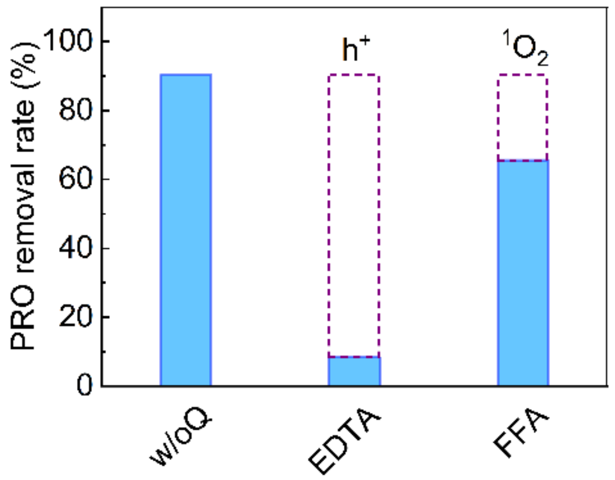

Supplementary Figure 19. Comparison of the PRO removal rates among different EMF tests without the quencher (denoted as w/oQ) or with different quencher (EDTA-2Na and FFA). The reduced portions marked with the dotted boxes indicate the amounts degraded by the quenched oxidants, thus revealing the contribution ratios of different oxidants to pollutant degradation.

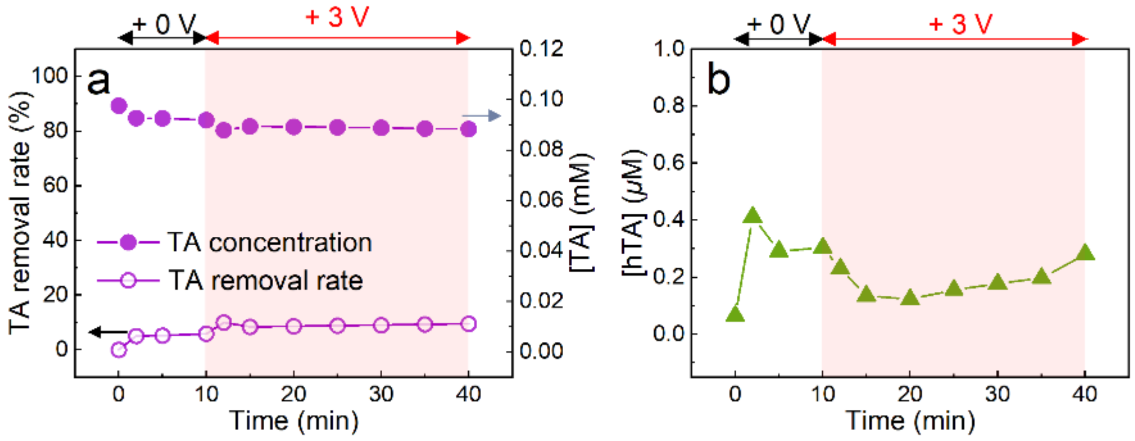

Supplementary Figure 20. Further revealing the mechanism of electrocatalytic degradation performance of the CN_900 membrane. (**a**) Probe test exploring the presence of ·OH through the degradation of 100 μM TA by the CN_900 membrane. (**b**) Variation of the measured concentration of hTA during the probe-test with TA for the CN_900 membrane.

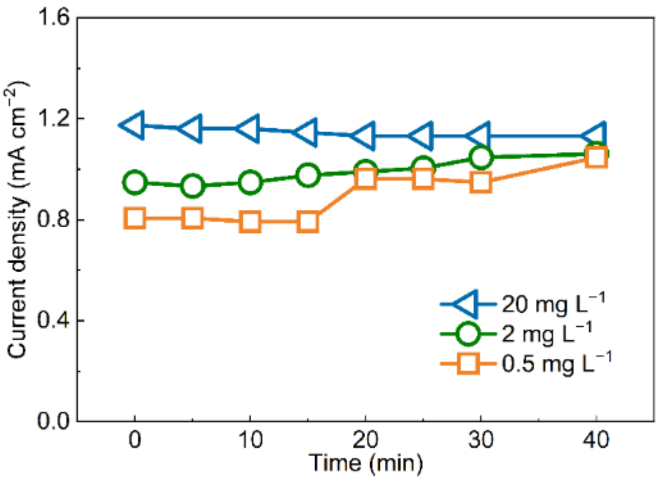

Supplementary Figure 21. Measured current densities (mA cm^{-2}) during the continuous EMF tests with the CN_900 membrane at different PRO concentrations (initial concentration: 20, 2, and 0.5 mg L^{-1}).

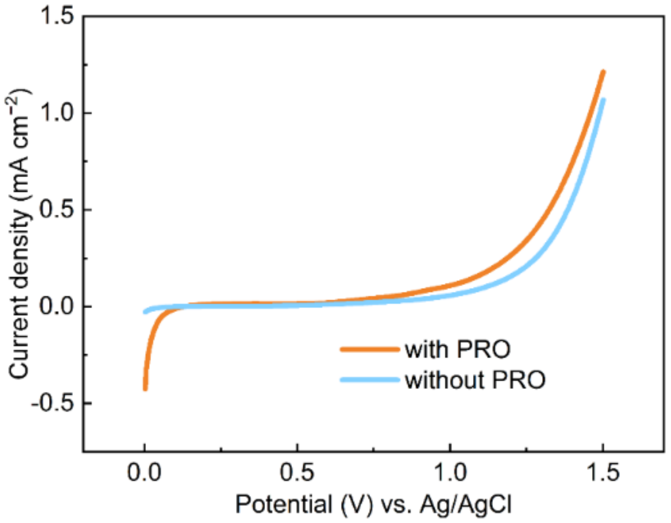

Supplementary Figure 22. Comparison of the LSV spectra between the measurements with and without the presence of PRO (20 mg L^{-1}) based on the CN_900 membrane.

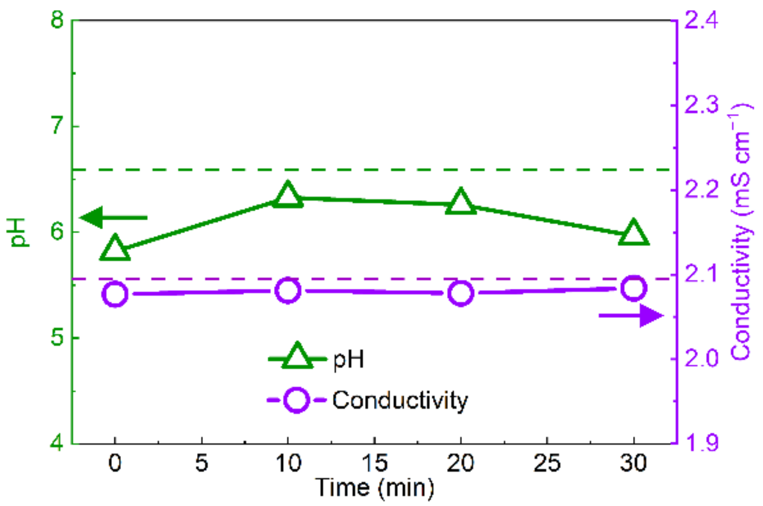

Supplementary Figure 23. Variations of the effluent pH and conductivity during the continuous EMF tests based on the CN_900 membrane and 20 mg L^{-1} PRO influent. The green and blue dotted lines indicate the influent pH and conductivity, respectively.

Supplementary Note 3.

Based on **Figure 6d** and **Figure 6g**, the kinetic degradation rate constants can be calculated for five different pollutants: 329.9 min^{-1} for RTD, 149.2 min^{-1} for CIP, 142.7 min^{-1} for CMT, 128.3 min^{-1} for SMX, and 114.1 min^{-1} for PRO. It can be seen that the reaction rate of direct anodic oxidation differs for different pollutants. RTD is more suitable for direct oxidation than the other four pollutants. The five pollutants (i.e., PRO, RTD, CIP, CMT, and SMX) used for the tests represent the most typical types of micropollutants of pharmaceuticals and personal care products that persist in water bodies: antibiotics (SMX and CIP), antagonists (CMT and RTD), and antihypertension (PRO). The reason we selected them is that they are difficult to degrade or remove by conventional water treatment technologies (e.g., biological processes, membrane bioreactors, etc.).

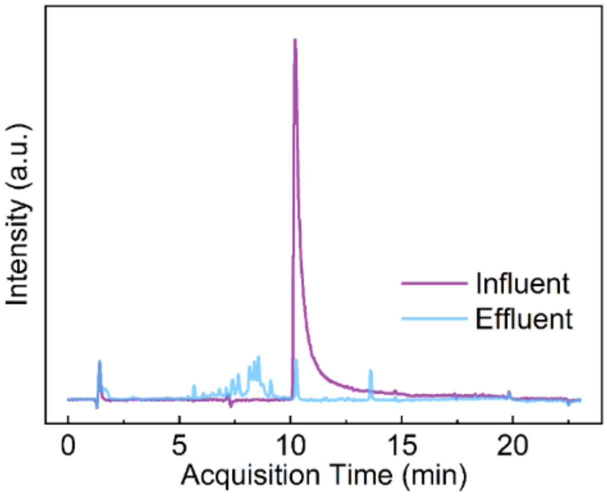

Supplementary Figure 24. Total ion current spectra measured by HPLC–MS/MS between the influent and effluent during the EMF tests based on the CN_900 membrane.

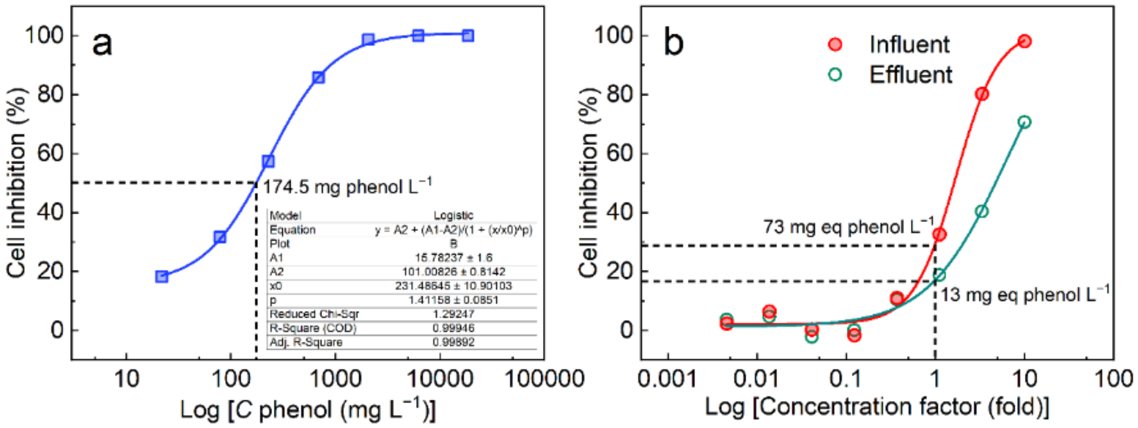

Supplementary Figure 25. (**a**) Standard curve of acute toxicity to *Vibrio fischeri* of phenol solution. The fitting parameters and EC50 values are shown in the figure. (**b**) Acute toxicity to *Vibrio fischeri* of the influent and effluent during the EMF tests based on the CN_900 membrane.

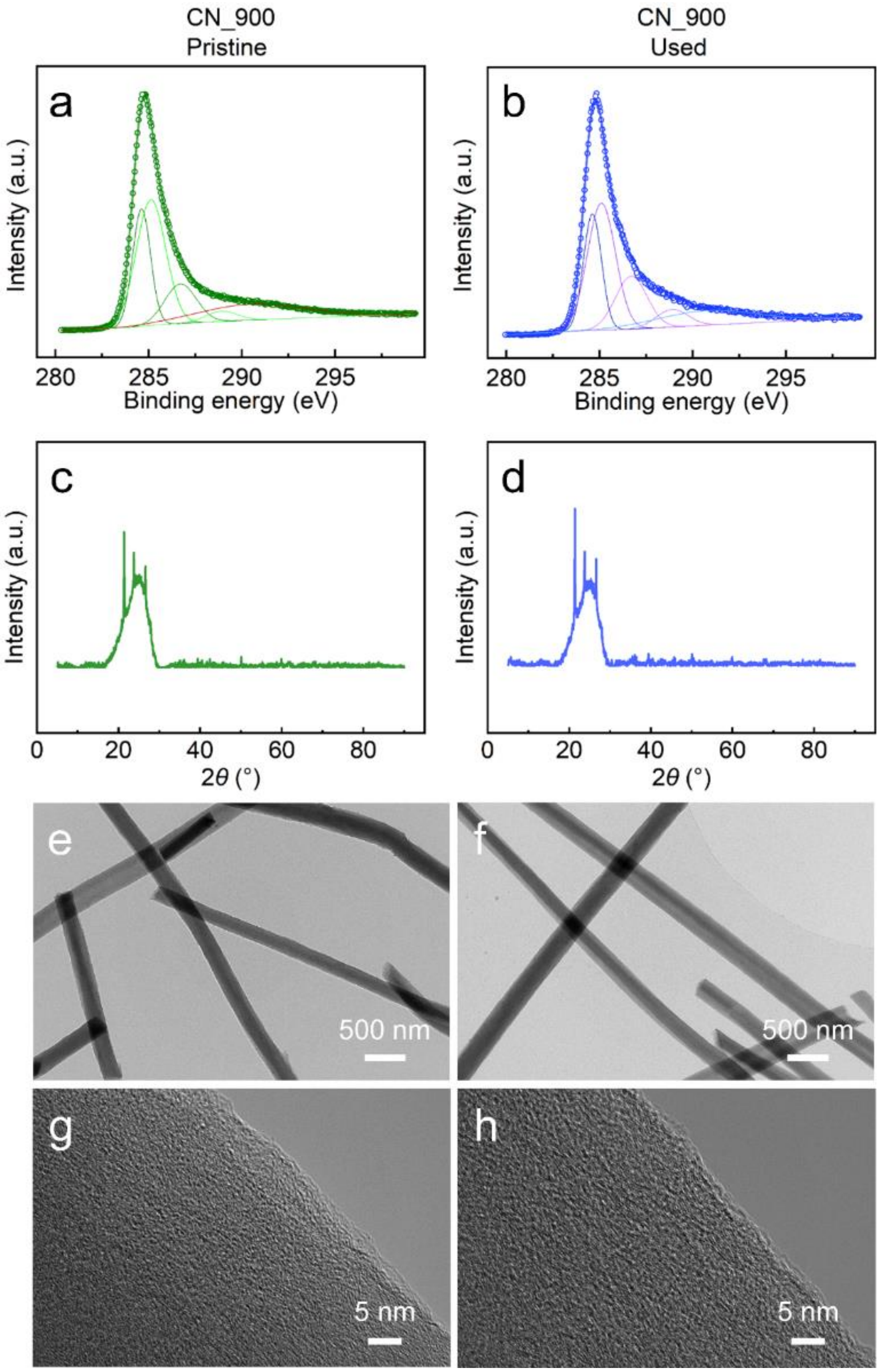

Supplementary Figure 26. Comparisons in terms of chemical compositions and morphology of the CN_900 between its pristine and long-term-used states. *C 1s* XPS spectra of the (**a**) pristine and (**b**) used CN_900. XRD spectra of the (**c**) pristine and (**d**) used CN_900. TEM images of the (**e**) pristine and (**f**) used CN_900. HRTEM images of the (**g**) pristine and (**h**) used CN_900.

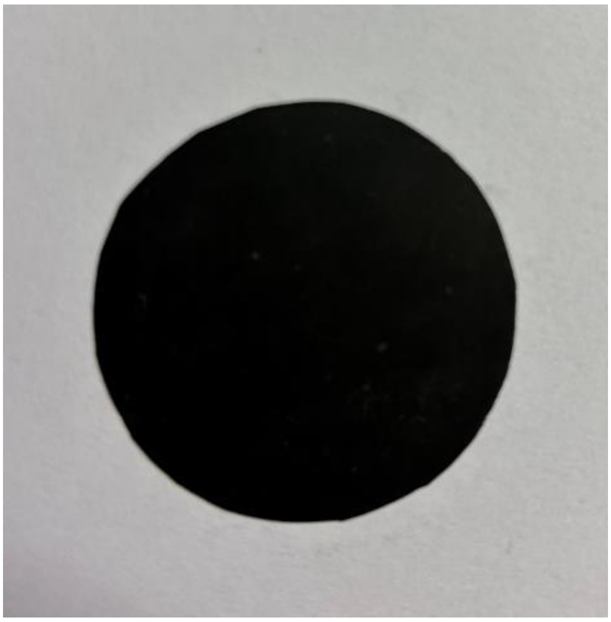

Supplementary Figure 27. Photograph of the CN_900 membrane after EMF process.

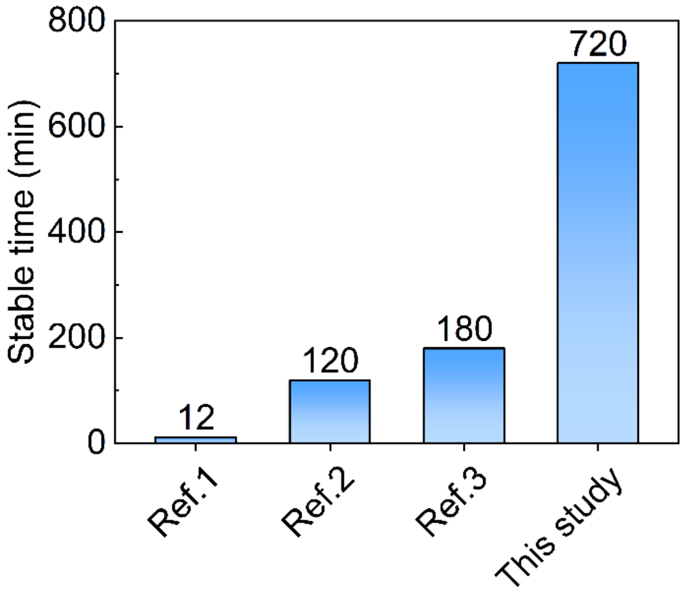

Supplementary Figure 28. Comparison of stable operation time between the CN_900 membrane in this study and other previously reported carbon-based electrocatalytic membranes (more details in **Supplementary Table 2**).

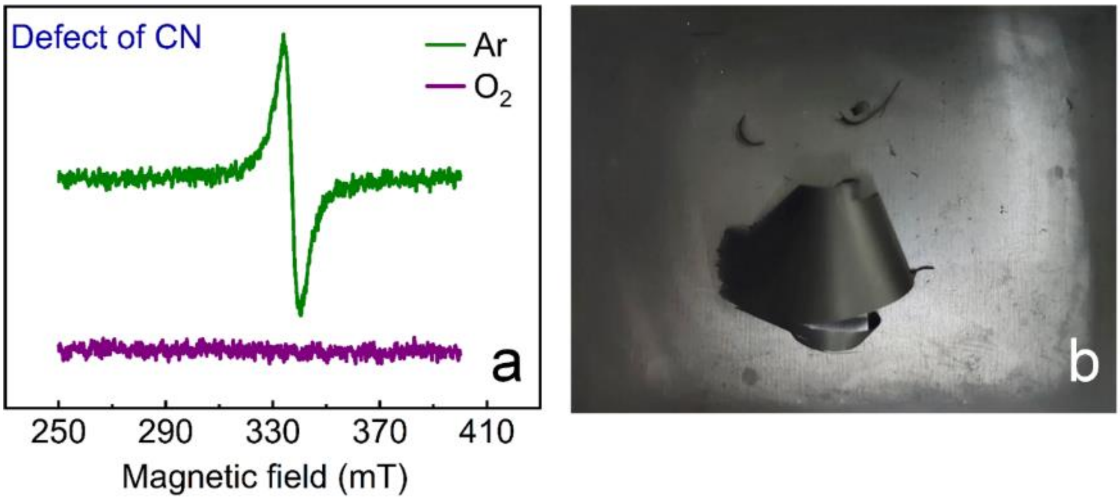

Supplementary Figure 29. (**a**) Comparison of ESR spectra of CN membranes carbonized at 900 °C under Ar and air atmospheres (with O_2). (**b**) Photographs of the CN membranes carbonized at 900 °C under air atmospheres (with O_2).

Supplementary Table 1. Calculated Hirshfeld charges, condensed Fukui functions and condensed dual descriptors (CDD) of CN_900.

Atom	q(N)	q(N+1)	q(N−1)	f^{-}	f^{+}	f^{0}	CDD
1(C)	0.029	-0.0015	0.0521	0.023	0.0305	0.0268	0.0075
2(C)	0.019	-0.0033	0.0466	0.0276	0.0224	0.025	-0.0052
3(C)	-0.0062	-0.0093	0.0004	0.0066	0.0032	0.0049	-0.0034
4(C)	0.0083	-0.0211	0.0351	0.0268	0.0295	0.0281	0.0026
5(C)	-0.0018	-0.009	0.0026	0.0044	0.0072	0.0058	0.0027
6(C)	0.0047	0.0006	0.009	0.0043	0.0041	0.0042	-0.0002
7(C)	-0.0058	-0.0143	0.0041	0.0099	0.0086	0.0092	-0.0014
8(C)	0.021	-0.0023	0.0512	0.0302	0.0233	0.0267	-0.0069
9(N)	-0.1572	-0.1807	-0.1363	0.0209	0.0235	0.0222	0.0026
10(C)	0.0089	-0.0076	0.026	0.0171	0.0165	0.0168	-0.0006
11(C)	0.0074	-0.0058	0.0185	0.0111	0.0132	0.0121	0.0021
12(C)	-0.0054	-0.0146	0.0062	0.0117	0.0092	0.0104	-0.0025
13(C)	0.0048	0.0005	0.0098	0.0051	0.0042	0.0046	-0.0008
14(C)	-0.0027	-0.011	0.0032	0.0059	0.0083	0.0071	0.0025
15(C)	0.0133	-0.0114	0.0312	0.0179	0.0246	0.0213	0.0067
16(C)	0.0064	0.0002	0.0122	0.0059	0.0062	0.006	0.0003
17(C)	-0.0018	-0.0128	0.0038	0.0056	0.0109	0.0083	0.0053
18(C)	0.0243	0.0092	0.0401	0.0158	0.0152	0.0155	-0.0006
19(C)	-0.0086	-0.0214	0.0058	0.0145	0.0128	0.0136	-0.0017
20(C)	0.0155	-0.0038	0.0466	0.0311	0.0193	0.0252	-0.0117
21(N)	-0.1585	-0.1869	-0.1333	0.0252	0.0285	0.0268	0.0033
22(C)	-0.0398	-0.0546	-0.0199	0.0199	0.0148	0.0174	-0.0051
23(C)	0.0033	-0.0068	0.0153	0.0121	0.0101	0.0111	-0.002
24(C)	-0.0367	-0.0499	-0.019	0.0177	0.0132	0.0154	-0.0045
25(C)	-0.0032	-0.0149	0.0039	0.0072	0.0116	0.0094	0.0044

26(C)	0.0046	-0.0012	0.0111	0.0065	0.0058	0.0062	-0.0007
27(C)	-0.0086	-0.021	0.0063	0.0148	0.0124	0.0136	-0.0024
28(C)	0.021	0.0085	0.0399	0.0189	0.0124	0.0157	-0.0065
29(N)	-0.1546	-0.1825	-0.1288	0.0258	0.0279	0.0269	0.0022
30(C)	0.0315	0.0154	0.0412	0.0097	0.0161	0.0129	0.0064
31(C)	0.0205	0.0017	0.0445	0.024	0.0188	0.0214	-0.0053
32(C)	0.0053	0.001	0.0087	0.0033	0.0043	0.0038	0.001
33(C)	0.0056	0.003	0.0092	0.0036	0.0026	0.0031	-0.001
34(N)	-0.1595	-0.184	-0.1367	0.0228	0.0245	0.0237	0.0018
35(C)	0.0289	-0.0005	0.0464	0.0176	0.0294	0.0235	0.0119
36(C)	-0.0642	-0.1074	-0.013	0.0512	0.0432	0.0472	-0.008
37(C)	0.027	-0.0061	0.057	0.03	0.0331	0.0316	0.0031
38(C)	-0.068	-0.0976	-0.0367	0.0313	0.0296	0.0304	-0.0017
39(C)	-0.0377	-0.0441	-0.0306	0.0071	0.0064	0.0068	-0.0007
40(C)	0.0034	-0.0187	0.0265	0.0231	0.022	0.0226	-0.0011
41(C)	-0.0041	-0.0101	0.0006	0.0047	0.006	0.0054	0.0012
42(N)	-0.1601	-0.1785	-0.1417	0.0184	0.0184	0.0184	-0.0001
43(C)	-0.05	-0.0548	-0.0453	0.0048	0.0047	0.0048	0
44(C)	0.0106	0.002	0.021	0.0104	0.0085	0.0095	-0.0019
45(C)	-0.0066	-0.03	0.0146	0.0212	0.0233	0.0223	0.0021
46(C)	-0.0113	-0.0185	-0.0043	0.007	0.0072	0.0071	0.0002
47(N)	-0.1604	-0.1924	-0.1303	0.0301	0.032	0.031	0.002
48(C)	0.0309	0.0248	0.0344	0.0034	0.0061	0.0048	0.0026
49(H)	0.0443	0.0326	0.0543	0.01	0.0117	0.0109	0.0017
50(H)	0.0422	0.0334	0.0516	0.0094	0.0089	0.0091	-0.0005
51(H)	0.0444	0.0332	0.0558	0.0115	0.0111	0.0113	-0.0003
52(H)	0.0425	0.0353	0.0502	0.0077	0.0072	0.0075	-0.0004
53(H)	0.0472	0.0328	0.0634	0.0162	0.0143	0.0153	-0.0019

54(H)	0.0511	0.0381	0.0648	0.0137	0.013	0.0133	-0.0007
55(H)	0.0491	0.0368	0.0621	0.013	0.0123	0.0126	-0.0006
56(H)	0.0436	0.0353	0.0528	0.0092	0.0083	0.0087	-0.0008
57(H)	0.0452	0.0372	0.052	0.0068	0.0079	0.0074	0.0011
58(H)	0.0437	0.0343	0.0535	0.0098	0.0095	0.0096	-0.0003
59(H)	0.0422	0.0298	0.0518	0.0097	0.0123	0.011	0.0027
60(H)	0.046	0.0263	0.0653	0.0193	0.0197	0.0195	0.0003
61(H)	0.0435	0.0311	0.0566	0.0131	0.0124	0.0127	-0.0008
62(H)	0.0411	0.028	0.0533	0.0122	0.0131	0.0126	0.0009
63(H)	0.048	0.0319	0.0639	0.0159	0.016	0.016	0.0001
64(H)	0.0446	0.0371	0.0523	0.0076	0.0075	0.0076	-0.0001
65(H)	0.0414	0.0327	0.0503	0.0089	0.0087	0.0088	-0.0002
66(H)	0.0409	0.0329	0.0489	0.008	0.008	0.008	0
67(H)	0.0397	0.0321	0.0472	0.0075	0.0076	0.0076	0.0002
68(H)	0.0366	0.0289	0.0439	0.0073	0.0077	0.0075	0.0004
69(H)	0.0372	0.0254	0.0486	0.0114	0.0118	0.0116	0.0003
70(H)	0.0433	0.038	0.0481	0.0048	0.0053	0.0051	0.0005

Supplementary Table 2. Calculated Hirshfeld charges, condensed Fukui functions and CDD of PRO.

Atom	q(N)	q(N+1)	q(N−1)	f^-	f^+	f^0	CDD
1(O)	-0.231	-0.2387	-0.2218	0.0093	0.0077	0.0085	-0.0015
2(C)	0.0463	0.0412	0.0529	0.0066	0.0051	0.0059	-0.0014
3(C)	-0.0142	-0.0172	0.0021	0.0163	0.0031	0.0097	-0.0132
4(N)	-0.1681	-0.1648	-0.0653	0.1028	-0.0033	0.0497	-0.1061
5(C)	0.0176	0.0156	0.0297	0.0121	0.002	0.0071	-0.0101
6(C)	-0.0987	-0.1018	-0.0783	0.0204	0.0031	0.0117	-0.0173
7(C)	-0.0896	-0.0914	-0.0815	0.0082	0.0018	0.005	-0.0064
8(C)	0.029	0.0218	0.0342	0.0052	0.0072	0.0062	0.002
9(O)	-0.0763	-0.0986	-0.0491	0.0273	0.0222	0.0248	-0.005
10(C)	0.0748	0.0076	0.1127	0.0379	0.0671	0.0525	0.0292
11(C)	-0.0786	-0.1313	-0.0336	0.045	0.0528	0.0489	0.0078
12(C)	-0.0471	-0.1132	-0.0015	0.0456	0.0661	0.0558	0.0205
13(C)	-0.0549	-0.1269	0.0157	0.0705	0.0721	0.0713	0.0015
14(C)	-0.0024	-0.0204	0.009	0.0114	0.018	0.0147	0.0066
15(C)	-0.0402	-0.1284	0.0121	0.0523	0.0882	0.0702	0.0359
16(C)	-0.0428	-0.1098	-0.002	0.0409	0.067	0.0539	0.0261
17(C)	-0.044	-0.1102	0.0013	0.0453	0.0662	0.0557	0.0209
18(C)	-0.0383	-0.1278	0.0002	0.0385	0.0895	0.064	0.0511
19(C)	-0.0146	-0.0327	-0.0066	0.008	0.0181	0.013	0.0101
20(H)	0.1595	0.1547	0.1714	0.0119	0.0048	0.0084	-0.0071
21(H)	0.0264	0.0119	0.0463	0.0199	0.0144	0.0172	-0.0055
22(H)	0.0359	0.0223	0.0596	0.0237	0.0136	0.0186	-0.0101
23(H)	0.0158	0.0115	0.0419	0.0261	0.0043	0.0152	-0.0218
24(H)	0.0754	0.0838	0.0912	0.0158	-0.0084	0.0037	-0.0242
25(H)	0.0283	0.0166	0.0505	0.0222	0.0117	0.0169	-0.0105

26(H)	0.0256	0.0147	0.0515	0.0259	0.0109	0.0184	-0.0149
27(H)	0.0245	0.0194	0.0367	0.0121	0.0051	0.0086	-0.007
28(H)	0.0267	0.0324	0.0337	0.007	-0.0057	0.0006	-0.0127
29(H)	0.0278	0.0157	0.0478	0.02	0.0121	0.0161	-0.0079
30(H)	0.0308	0.0288	0.0411	0.0103	0.0019	0.0061	-0.0083
31(H)	0.0272	0.0358	0.0284	0.0012	-0.0086	-0.0037	-0.0099
32(H)	0.0342	0.0237	0.0453	0.0111	0.0106	0.0108	-0.0005
33(H)	0.0324	0.0219	0.0483	0.0159	0.0105	0.0132	-0.0054
34(H)	0.0373	0.0073	0.0595	0.0222	0.03	0.0261	0.0078
35(H)	0.0447	0.0053	0.0735	0.0288	0.0394	0.0341	0.0105
36(H)	0.04	0.0025	0.0711	0.031	0.0375	0.0343	0.0065
37(H)	0.0434	0.0012	0.0698	0.0263	0.0423	0.0343	0.0159
38(H)	0.0447	0.0039	0.0718	0.0271	0.0408	0.034	0.0137
39(H)	0.0459	0.0056	0.0715	0.0256	0.0403	0.0329	0.0147
40(H)	0.0467	0.0082	0.0592	0.0125	0.0385	0.0255	0.026

Supplementary Table 3. The referenced studies in the comparison of electrocatalysis efficiency.

	Membrane materials	k, min^{-1}	1/EEO, m^3 order kWh^{-1}	Reference
	N-doped defective carbon nanofiber	114.1	34.48	This study
S1	BDD/ADE–Pt/CF	0.0029	0.015	[5]
S2	Nb/BDD	0.0101	0.024	[6]
S3	Ti/$Ir_xTa_yO_2$/$[Bi_2O_3]_z[TiO_2]_{1-z}$	0.0217	0.208	[7]
S4	Ti/Pt	0.0326	0.227	[8]
S5	Ti-Pt	0.0226	0.571	[9]
S6	Pt	0.9210	3.030	[10]
S7	Pd–Pt–ceramic membrane	4.6072	57.67	[11]
S8	Cobalt-doped Black-TiO_2 nanotube array	0.0048	0.011	[12]
S9	Ti/$Ru_{0.3}Ti_{0.7}O$	0.0161	0.016	[13]
S10	Electrodeposited polytetrafluoroethylene (PTFE)-doped β-PbO_2	0.0154	0.020	[14]
S11	3G/SnO_2/CFs	0.238	0.024	[15]
S12	A composite wire mesh anode (composed of blue TiO_2 nanotubes covered with SnO_2–Sb_2O_3)	0.0076	0.121	[16]
S13	TiO_2 nanotubes arrays electrode	0.0253	0.122	[17]
S14	Sb-doped SnO_2 with TiO_2 nanotube clusters on Ti mesh	0.0676	0.312	[18]
S15	Ti/SnO_2-Sb	0.24	0.690	[19]
S16	TiO_2@SnO_2–Sb	0.0019	0.805	[20]
S17	Ti/SnO_2-Sb/Ce-PbO_2	0.649	1.344	[21]
S18	Blue-TiO_2 nanotubes	0.0071	1.587	[22]
S19	Macroporous-Ti-enhanced TiO_2 nanotube	0.124	1.724	[23]

	array/SnO_2-Sb_2O_3			
S20	Bi-doped SnO_2–Ti_nO_{2n-1}	115.1	2.381	[24]
S21	SnO_2-Sb reactive anodic filter	1.485	3.030	[25]
S22	Ti_4O_7	20.98	3.704	[26]
S23	Blue-colored TiO_2 nanotube arrays	0.403	11.63	[27]
S24	Ti_4O_7 reactive electrochemical membrane	0.0298	4.303	[28]
S25	Porous Ti-ENTA/SnO_2-Sb anode	0.1197	0.049	[29]
S26	Flow-through Ti/TiO_2-NTA/Ti_4O_7 anode	0.083	0.758	[30]
S27	TiSO electroactive ceramic membrane	0.124	0.235	[31]
S28	Reduced TiO_2 nanotube arrays-based Ti membrane	0.0395	1.818	[32]
S29	Porous Ti–ENTA/SnO_2–Sb	0.0914	1.613	[33]
S30	IrO_2 and Ta_2O_5 coating on titanium mesh and Fe foam coated with Pd	0.0894	0.019	[34]
S31	Blue TiO_2 nanotube electrocatalytic membrane	0.235	3.704	[35]
S32	Multi-walled carbon nanotube	2.1	0.500	[36]
S33	Carbon nanotube electrochemical filter	98.9	6.933	[37]
S34	Coal-based carbon membrane	3.985	20.31	[38]
S35	FeNi LDH/CNT membranes	0.0328	2.580	[39]
S36	Graphene sponge	1.002	0.149	[40]
S37	FeOCl-CNT filters	106.0	6.848	[41]
S38	CNT filter modified with MIL-101(Fe)	0.015	7.297	[42]
S39	BDD	0.0388	0.005	[43]
S40	BDD	0.0158	0.040	[44]
S41	BDD	0.0715	1.429	[45]

Supplementary Table 4. The referenced studies in the comparison of stable operation time in **Supplementary Figure 28**.

Reference	Electrocatalyst	Stable time, min	Target pollutant
[46]	Hybrid graphene-decorated metal hollow fiber membrane	12	Paracetamol
[36]	Multi-walled carbon nanotube filter	120	Sulfamethoxazole
[37]	Carbon nanotube electrochemical filter	180	Tetracycline
This study	N-doped defective carbon nanofiber membrane	720	Propranolol

Supplementary Table 5. The boundary condition and related coefficients used in the multiphysics simulations.

Parameter	Value	Definition
μ_in	3×10^{-4} m s^{-1}	flow-in velocity
n	1	number of electrons transferred
a	0.5	electron transfer coefficient
E^0	2.5 V	formal potential
E	3 V	applied potential
i_0	8×10^{-4} A m^{-2}	exchange current density
C_0	0.0676 mol m^{-3}	pollutant concentration at inlet
κ_s	0.22 S m^{-1}	conductivity of solution
D	5×10^{-10} m^2 s^{-1}	pollutant diffusivity
T	293.15 K	room temperature

References

1. Melis N, Mocci F, Vacca A & Pilia L. Novel homogeneous selective electrocatalysts for CO_2 reduction: an electrochemical and computational study of cyclopentadienyl-phenylendiamino-cobalt complexes. *Sustainable Energy Fuels* **4**, 5609–5617 (2020).

2. Duan X, *et al.* Electrocatalytic degradation of 2,4-dichlorophenol by a 3DG-PbO_2 powdered anode: Experimental and theoretical insights. *Sep Purif Technol* **282**, 120003 (2022).
3. Lu T & Chen F. Multiwfn: A multifunctional wavefunction analyzer. *J Comput Chem* **33**, 580–592 (2012).
4. Lu T & Chen Q. Realization of conceptual density functional theory and information-theoretic approach in Multiwfn program. In: *Conceptual Density Functional Theory*) (2022).
5. Isarain-Chávez E, *et al.* Degradation of pharmaceutical beta-blockers by electrochemical advanced oxidation processes using a flow plant with a solar compound parabolic collector. *Water Res* **45**, 4119–4130 (2011).
6. García-Espinoza JD, Zolfaghari M & Mijaylova Nacheva P. Synergistic effect between ultraviolet irradiation and electrochemical oxidation for removal of humic acids and pharmaceuticals. *Water Environ J* **34**, 232–246 (2020).
7. Jasper JT, Shafaat OS & Hoffmann MR. Electrochemical transformation of trace organic contaminants in latrine wastewater. *Environ Sci Technol* **50**, 10198–10208 (2016).
8. Song H, *et al.* Nonradical oxidation from electrochemical activation of peroxydisulfate at Ti/Pt anode: Efficiency, mechanism and influencing factors. *Water Res* **116**, 182–193 (2017).
9. Zambrano J & Min B. Comparison on efficiency of electrochemical phenol oxidation in two different supporting electrolytes (NaCl and Na_2SO_4) using Pt/Ti electrode. *Environ Technol Innovation* **15**, 100382 (2019).
10. Mbaye M, *et al.* Rapid removal of fungicide thiram in aqueous medium by electro-Fenton process with Pt and BDD anodes. *Sep Purif Technol* **281**, 119837 (2022).
11. Zhao Y, *et al.* Janus electrocatalytic flow-through membrane enables highly selective singlet oxygen production. *Nat Commun* **11**, 6228 (2020).
12. Yang Y, *et al.* Cobalt-doped black TiO_2 nanotube array as a stable anode for oxygen evolution and electrochemical wastewater treatment. *ACS Catal* **8**, 4278–4287 (2018).
13. Fukunaga MT, Guimarães JR & Bertazzoli R. Kinetics of the oxidation of formaldehyde in a flow electrochemical reactor with TiO_2/RuO_2 anode. *Chem Eng J* **136**, 236–241 (2008).
14. Sánchez-Montes I, *et al.* Evolution of the antibacterial activity and oxidation intermediates during the electrochemical degradation of norfloxacin in a flow cell with a PTFE-doped β-PbO_2 anode: Critical comparison to a BDD anode. *Electrochim Acta* **284**, 260–270 (2018).
15. Yu S, *et al.* Electrospun PAN-based graphene/SnO_2 carbon nanofibers as anodic electrocatalysis microfiltration membrane for sulfamethoxazole degradation. *J Membr Sci* **614**, 118368 (2020).
16. Meng X, *et al.* Development of a three-dimensional electrochemical system using a blue TiO_2/SnO_2–Sb_2O_3 anode for treating low-ionic-strength wastewater. *Environ Sci Technol* **53**, 13784–13793 (2019).

17. Wang K, *et al.* Insights into electrochemical decomposition mechanism of lipopolysaccharide using TiO_2 nanotubes arrays electrode. *J Hazard Mater* **391**, 122259 (2020).

18. Huang L, *et al.* Construction of TiO_2 nanotube clusters on Ti mesh for immobilizing Sb-SnO_2 to boost electrocatalytic phenol degradation. *J Hazard Mater* **393**, 122329 (2020).

19. Zhou C, Wang Y, Chen J & Niu J. Porous Ti/SnO_2-Sb anode as reactive electrochemical membrane for removing trace antiretroviral drug stavudine from wastewater. *Environ Int* **133**, 105157 (2019).

20. Zheng J, Wang Z, Ma J, Xu S & Wu Z. Development of an electrochemical ceramic membrane filtration system for efficient contaminant removal from waters. *Environ Sci Technol* **52**, 4117–4126 (2018).

21. Xu L, Ma X, Niu J, Chen J & Zhou C. Removal of trace naproxen from aqueous solution using a laboratory-scale reactive flow-through membrane electrode. *J Hazard Mater* **379**, 120692 (2019).

22. Cai J, Zhou M, Pan Y, Du X & Lu X. Extremely efficient electrochemical degradation of organic pollutants with co-generation of hydroxyl and sulfate radicals on Blue-TiO_2 nanotubes anode. *Appl Catal B* **257**, 117902 (2019).

23. Chen M, *et al.* Development of a highly efficient electrochemical flow-through anode based on inner in-site enhanced TiO_2-nanotubes array. *Environ Int* **140**, 105813 (2020).

24. Gayen P, Chen C, Abiade JT & Chaplin BP. Electrochemical oxidation of atrazine and clothianidin on Bi-doped SnO_2–Ti_nO_{2n-1} electrocatalytic reactive electrochemical membranes. *Environ Sci Technol* **52**, 12675–12684 (2018).

25. Yang C, Fan Y, Shang S, Li P & Li X-y. Fabrication of a permeable SnO_2-Sb reactive anodic filter for high-efficiency electrochemical oxidation of antibiotics in wastewater. *Environ Int* **157**, 106827 (2021).

26. Misal SN, Lin M-H, Mehraeen S & Chaplin BP. Modeling electrochemical oxidation and reduction of sulfamethoxazole using electrocatalytic reactive electrochemical membranes. *J Hazard Mater*, 121420 (2019).

27. Xu L, *et al.* Effective degradation of aqueous carbamazepine on a novel blue-colored TiO_2 nanotube arrays membrane filter anode. *J Hazard Mater* **402**, 123530 (2021).

28. Zhi D, *et al.* Development of ozonation and reactive electrochemical membrane coupled process: Enhanced tetracycline mineralization and toxicity reduction. *Chem Eng J* **383**, 123149 (2020).

29. Hakizimana I, *et al.* Effective degradation of amoxicillin by multi-stage flow-through electrochemical system using porous electrodes. *Sep Purif Technol* **300**, 121832 (2022).

30. Meng C, *et al.* Efficient electrochemical oxidation of COVID-19 treatment drugs favipiravir by a novel flow-through Ti/TiO_2-NTA/Ti_4O_7 anode. *Electrochim Acta* **430**, 141055 (2022).

31. Wang S, Pei S, Zhang J, Huang J & You S. Flow-through electrochemical removal of benzotriazole by electroactive ceramic membrane. *Water Res* **218**, 118454 (2022).

32. Zeng W, *et al.* Efficient electrochemical oxidation of sulfamethoxazole by a novel

reduced TiO_2 nanotube arrays-based flow-through electrocatalytic membrane. *Sep Purif Technol* **289**, 120720 (2022).

33. Zhang C, Zhao X, Wang C & Pan S. Novel multistage electrochemical flow-through mode (EFTM) with porous electrodes for reclaimed wastewater treatment in pipes. *ACS ES&T Water* **1**, 653–660 (2021).
34. Hyldegaard BH, Ottosen LM & Alshawabkeh AN. Transformation of tetrachloroethylene in a flow-through electrochemical reactor. *Sci Total Environ* **707**, 135566 (2020).
35. Wang L*, et al.* Blue TiO_2 nanotube electrocatalytic membrane electrode for efficient electrochemical degradation of organic pollutants. *Chemosphere* **306**, 135628 (2022).
36. Tan T-Y*, et al.* Electrochemically enhanced simultaneous degradation of sulfamethoxazole, ciprofloxacin and amoxicillin from aqueous solution by multi-walled carbon nanotube filter. *Sep Purif Technol* **235**, 116167 (2020).
37. Liu Y*, et al.* Degradation of the common aqueous antibiotic tetracycline using a carbon nanotube electrochemical filter. *Environ Sci Technol* **49**, 7974–7980 (2015).
38. Pan Z*, et al.* Electrochemical microfiltration treatment of bisphenol A wastewater using coal-based carbon membrane. *Sep Purif Technol* **227**, 115695 (2019).
39. Yang L, Xu D, Yang H, Luo X & Liang H. Structurally-controlled FeNi LDH/CNTs electro-Fenton membrane for in-situ electro-generation and activation of hydroxyl radicals toward organic micropollutant treatment. *Chem Eng J* **432**, 134436 (2022).
40. Ormeno-Cano N & Radjenovic J. Electrochemical degradation of antibiotics using flow-through graphene sponge electrodes. *J Hazard Mater* **431**, 128462 (2022).
41. Li Z*, et al.* Carbon nanotube filter functionalized with iron oxychloride for flow-through electro-Fenton. *Appl Catal B* **260**, 118204 (2020).
42. Dai Y*, et al.* Carbon nanotube filter functionalized with MIL-101(Fe) for enhanced flow-through electro-Fenton. *Environ Res* **204**, 112117 (2022).
43. Carneiro JF*, et al.* The effect of the supporting electrolyte on the electrooxidation of enrofloxacin using a flow cell with a BDD anode: Kinetics and follow-up of oxidation intermediates and antimicrobial activity. *Chemosphere* **206**, 674–681 (2018).
44. Coledam DAC, Aquino JM, Silva BF, Silva AJ & Rocha-Filho RC. Electrochemical mineralization of norfloxacin using distinct boron-doped diamond anodes in a filter-press reactor, with investigations of toxicity and oxidation by-products. *Electrochim Acta* **213**, 856–864 (2016).
45. Carneiro JF, Aquino JM, Silva BF, Silva AJ & Rocha-Filho RC. Comparing the electrochemical degradation of the fluoroquinolone antibiotics norfloxacin and ciprofloxacin using distinct electrolytes and a BDD anode: evolution of main oxidation byproducts and toxicity. *J Environ Chem Eng* **8**, 104433 (2020).
46. Huong Le TX*, et al.* Hybrid graphene-decorated metal hollow fibre membrane reactors for efficient electro-Fenton - Filtration co-processes. *J Membr Sci* **587**, 117182 (2019).
